# Engineering of a DNA/γPNA Hybrid Nanoreporter for ctDNA Mutation Detection via γPNA Urinalysis

**DOI:** 10.1002/advs.202310225

**Published:** 2024-07-03

**Authors:** Zhichu Xiang, Jianhua Lu, Yang Ming, Weisheng Guo, Xiaoyuan Chen, Weijian Sun

**Affiliations:** ^1^ Department of Gastrointestinal Surgery The Second Affiliated Hospital and Yuying Children's Hospital of Wenzhou Medical University Wenzhou Medical University Wenzhou 325027 China; ^2^ Departments of Diagnostic Radiology, Surgery, Chemical and Biomolecular Engineering, and Biomedical Engineering Yong Loo Lin School of Medicine and College of Design and Engineering National University of Singapore Singapore 119074 Singapore; ^3^ Department of Minimally Invasive Interventional Radiology The State Key Laboratory of Respiratory Disease School of Biomedical Engineering & The Second Affiliated Hospital Guangzhou Medical University Guangzhou 510260 China; ^4^ Clinical Imaging Research Centre Centre for Translational Medicine Yong Loo Lin School of Medicine National University of Singapore Singapore 117599 Singapore; ^5^ Nanomedicine Translational Research Program Yong Loo Lin School of Medicine National University of Singapore Singapore 117597 Singapore; ^6^ Theranostics Center of Excellence (TCE) Yong Loo Lin School of Medicine National University of Singapore 11 Biopolis Way, Helios Singapore 138667 Singapore; ^7^ Institute of Molecular and Cell Biology Agency for Science, Technology, and Research (A*STAR) 61 Biopolis Drive, Proteos Singapore 138673 Singapore

**Keywords:** ctDNA mutation, tumor detection, urinalysis, γPNA

## Abstract

Detection of circulating tumor DNA (ctDNA) mutations, which are molecular biomarkers present in bodily fluids of cancer patients, can be applied for tumor diagnosis and prognosis monitoring. However, current profiling of ctDNA mutations relies primarily on polymerase chain reaction (PCR) and DNA sequencing and these techniques require preanalytical processing of blood samples, which are time‐consuming, expensive, and tedious procedures that increase the risk of sample contamination. To overcome these limitations, here the engineering of a DNA/γPNA (gamma peptide nucleic acid) hybrid nanoreporter is disclosed for ctDNA biosensing via in situ profiling and recording of tumor‐specific DNA mutations. The low tolerance of γPNA to single mismatch in base pairing with DNA allows highly selective recognition and recording of ctDNA mutations in peripheral blood. Owing to their remarkable biostability, the detached γPNA strands triggered by mutant ctDNA will be enriched in kidneys and cleared into urine for urinalysis. It is demonstrated that the nanoreporter has high specificity for ctDNA mutation in peripheral blood, and urinalysis of cleared γPNA can provide valuable information for tumor progression and prognosis evaluation. This work demonstrates the potential of the nanoreporter for urinary monitoring of tumor and patient prognosis through in situ biosensing of ctDNA mutations.

## Graphical abstract

A DNA/γPNA hybrid nanoreporter is engineered for urinalysis of tumor‐specific ctDNA mutations in peripheral blood with high selectivity. The specific recognition and recording of ctDNA mutations are accomplished by introducing γPNA into a DNA‐based toehold‐mediated strand displacement system. This work demonstrates the feasibility of employing nanoreporter for tumor urinalysis through in situ sensing and recording of ctDNA mutations in peripheral blood.

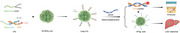

## 1 Introduction

Circulating tumor DNA (ctDNA) refers to the cell‐free DNA (cfDNA) strands with the length of less than 145 bp that is released into peripheral blood from apoptotic or necrotic cancerous cells during tumorigenesis, progression, and metastasis.^[^ ^1^, ^2^ ^]^ It is emerging as a predictive or prognostic biomarker of tumor progression and prognosis due to its excellent correlation with the molecular pathology of tumor.^[^ ^2^, ^3^, ^4^ ^]^ The ctDNA detection‐based liquid biopsy can not only provide valuable information on tumor stages but also noninvasively monitor the antitumor therapy efficacy.^[^ ^2^, ^5^ ^]^ Currently, the techniques for ctDNA profiling rely primarily on PCR and DNA sequencing in clinic applications.^[^ ^6^, ^7^ ^]^ PCR‐based techniques such as drop digital PCR (ddPCR) has been extensively investigated and turned out to be effective for point mutation detection of ctDNA in plasma. However, it is time‐consuming and the droplets production for ddPCR are difficult, which makes the clinical application of ddPCR challenging.^[^ ^8^ ^]^ DNA sequencing‐based technique such as next‐generation sequencing (NGS) is highly sensitive and reliable for ctDNA mutation profiling.^[^ ^9^ ^]^ Unfortunately, the clinical implementation of DNA sequencing is limited due to the high cost and the time frame for sequence information acquiring is long (2‐3 weeks).^[^ ^6^ ^]^ Moreover, the detection techniques based on PCR or DNA sequencing require blood sampling and preanalytical processing, which increase the risk of sample contamination and make the analysis complicated considering the short half‐life and low concentration of ctDNA in peripheral blood.^[^ ^1^, ^10^ ^]^

Peptide nucleic acids (PNAs) are a class of artificial DNA mimics that consist of natural nucleobases attached to the pseudopeptide backbone.^[^ ^11^, ^12^ ^]^ They can selectively recognize and bind to complementary DNA or RNA strands through Watson‐Crick base‐pairing with much higher thermal stability than DNA or RNA homoduplex due to the electrically neutral polyamide backbone of PNA.^[^ ^13^, ^14^ ^]^ Capitalizing on the high programmability and biocompatibility, PNA and DNA have been employed as biocompatible building blocks for the engineering of diverse nanoplatforms for various biomedical applications including biosensing,^[^ ^15^, ^16^, ^17^ ^]^ imaging,^[^ ^18^, ^19^, ^20^ ^]^ and stimuli‐responsive cargo delivery and release.^[^ ^21^, ^22^, ^23^ ^]^ In particular, to simplify the experiment procedure and improve the detection specificity and sensitivity, diverse DNA‐ and PNA‐based probes have recently been developed for ctDNA detection in plasma through electrochemical sensing,^[^ ^24^, ^25^, ^26^, ^27^ ^]^ surface plasmon resonance imaging (SPRI)^[^ ^28^, ^29^, ^30^ ^]^ and fluorogenic detection.^[^ ^31^, ^32^, ^33^, ^34^ ^]^ Despite the progress made of these probes in ctDNA biosensing, most of them require blood sampling and preanalytical processing of blood samples. Moreover, the production of sensors on the surface of a microelectronic chip for ctDNA sensing based on electrochemical sensing is not easy, and the detection through SPRI requires the preparation and further modification of gold chips and nanoparticles in multiple steps.

**Figure 1:**
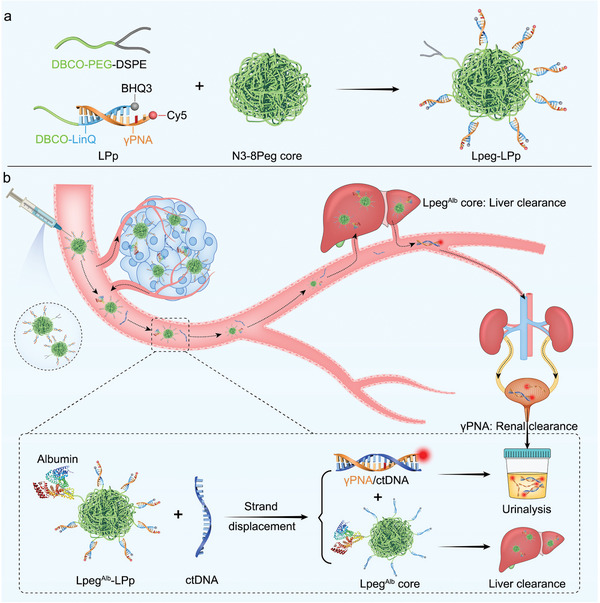
a) Schematic depiction of Lpeg‐LPp engineered for ctDNA detection. b) ctDNA mutation‐induced dissociation of γPNA from Lpeg‐LPp and clearance through kidneys to urine enable ctDNA mutation detection via γPNA urinalysis.

To overcome these challenges, herein we report the engineering of a DNA/gamma‐modified PNA (γPNA) hybrid nanoreporter (termed as Lpeg‐LPp) for ctDNA detection via in situ profiling and recording of tumor‐specific mutations in peripheral blood (**Figure** **1**). Specifically, a fluorophore cyanine 5 (Cy5)‐labeled γPNA oligomer was employed and hybridized with DNA to obtain a DNA/γPNA duplex. The fluorescence of the obtained duplex (LPp) was quenched by Black Hole Quencher 3 (BHQ3) labeled at 3′‐end of linker DNA (LinQ) via Föster resonance energy transfer (FRET),^[^ ^35^ ^]^ which involves the energy transfer from Cy5 to the nearby acceptor quencher when they are brought in proximity after the Cy5‐labeled γPNA is hybridized with LinQ. Then, the dibenzocyclooctyne (DBCO)‐bearing LPp duplex and 1,2‐distearoyl‐sn‐glycero‐3‐phosphoethanolamine‐N‐[amino(polyethylene glycol)−2000] (DSPE‐PEG, 2 kDa) were efficiently functionalized onto an 8‐arm poly(ethylene glycol) nanoparticle bearing azido groups on the arms (8PEG‐N3, 40 kDa) via click chemistry to obtain the nanoreporter Lpeg‐LPp.^[^ ^36^, ^37^ ^]^ PEG has good biosafety profiles and has been approved by the U.S. FDA for many biomedical applications.^[^ ^38^ ^]^ Moreover, the nanostructured PEG has longer circulation half‐life in peripheral blood and can prevent surface‐ligated LPps from filtering into urine before being triggered by ctDNA mutation, and the 8‐armed structure allows multivalent presentation of LPp. The low tolerance of γPNA to single‐base mismatch in hybridization with DNA enables the recognition of ctDNA mutations in peripheral blood with superior sensitivity and specificity.^[^ ^39^ ^]^ Although the γPNA for DNA/γPNA hybrid nanoreporter engineering has many advantages, it is not easy to synthesize and is more expensive than DNA. The albumin‐binding capability of diacyl lipid moiety contributes to the prolonged blood circulation half‐life of Lpeg‐LPp.^[^ ^40^ ^]^ Moreover, the enzyme‐resistant property of γPNA enables good biostability of the nanoreporter and contributes to durable monitoring and recording of ctDNA mutations in peripheral blood. In the presence of ctDNA with point mutations, it will specifically outcompete the γPNA strand of the duplex on Lpeg‐LPp to form a better‐matched duplex via toehold‐mediated strand displacement reaction which involves the toehold association, branch migration, and strand dissociation, leading to the detachment of γPNA from the nanoreporter and to be cleared from kidneys to urine.^[^ ^41^ ^]^ Notably, the larger hydrodynamic diameter of the PEG nanocore (≈10 nm) than the glomerulus pore size (≈5 nm) prevented the γPNA from being filtered into urine before the dissociation triggered by mutant ctDNA. Therefore, the γPNA strand will be detached from Lpeg‐LPp only in the presence of ctDNA mutations and further cleared through the kidneys to urine, enabling ctDNA mutation detection using the nanoreporter with no need for blood sampling and preanalytical processing.

## 2 Results and Discussion

### 2.1 Design and Engineering of Lpeg‐LPp

We chose proto‐oncogene KRAS(G12D) mutation (termed as 12DM), which is associated with many cancers and has been detected in the majority of pancreatic cancer patients (≈90%),^[^ ^42^, ^43^ ^]^ as target ctDNA to validate the feasibility of our design. A 12DM‐specific γPNA oligomer (Pp) was designed and hybridized with a linker DNA to construct the toehold‐bearing molecular reporter LPp (Table S1, Supporting Information). In vitro fluorogenic assay showed that the Cy5 signal of LPp was significantly quenched compared to Pp, indicating that the γPNA was successfully hybridized with linker DNA to form molecular reporter LPp (Figure S1, Supporting Information). To figure out whether the reporter could be used to detect ctDNA mutation, LPp was treated with 12DM, wild‐type (WT) oligonucleotide, and control DNA with random nucleobase sequence (CDNA) as control. As shown in Figure S1b (Supporting Information), a significant fluorescence increase could be observed when LPp was treated with 12DM, while the signal remained at background level after WT or CDNA treatment, demonstrating specific discrimination of 12DM. After confirming that the LPp could be used for ctDNA mutation detection, the molecular reporter was further ligated onto an 8‐arm PEG core to obtain the nanoreporter Lpeg‐LPp. The successful engineering of Lpeg‐LPp was confirmed by agarose gel electrophoresis assay (Figure S2a, Supporting Information), and the ratio between DSPE and LPp on the nanoreporter was validated according to the linear relationship between fluorescence intensity and concentration (Figure S2b, Supporting Information).

**Figure 2:**
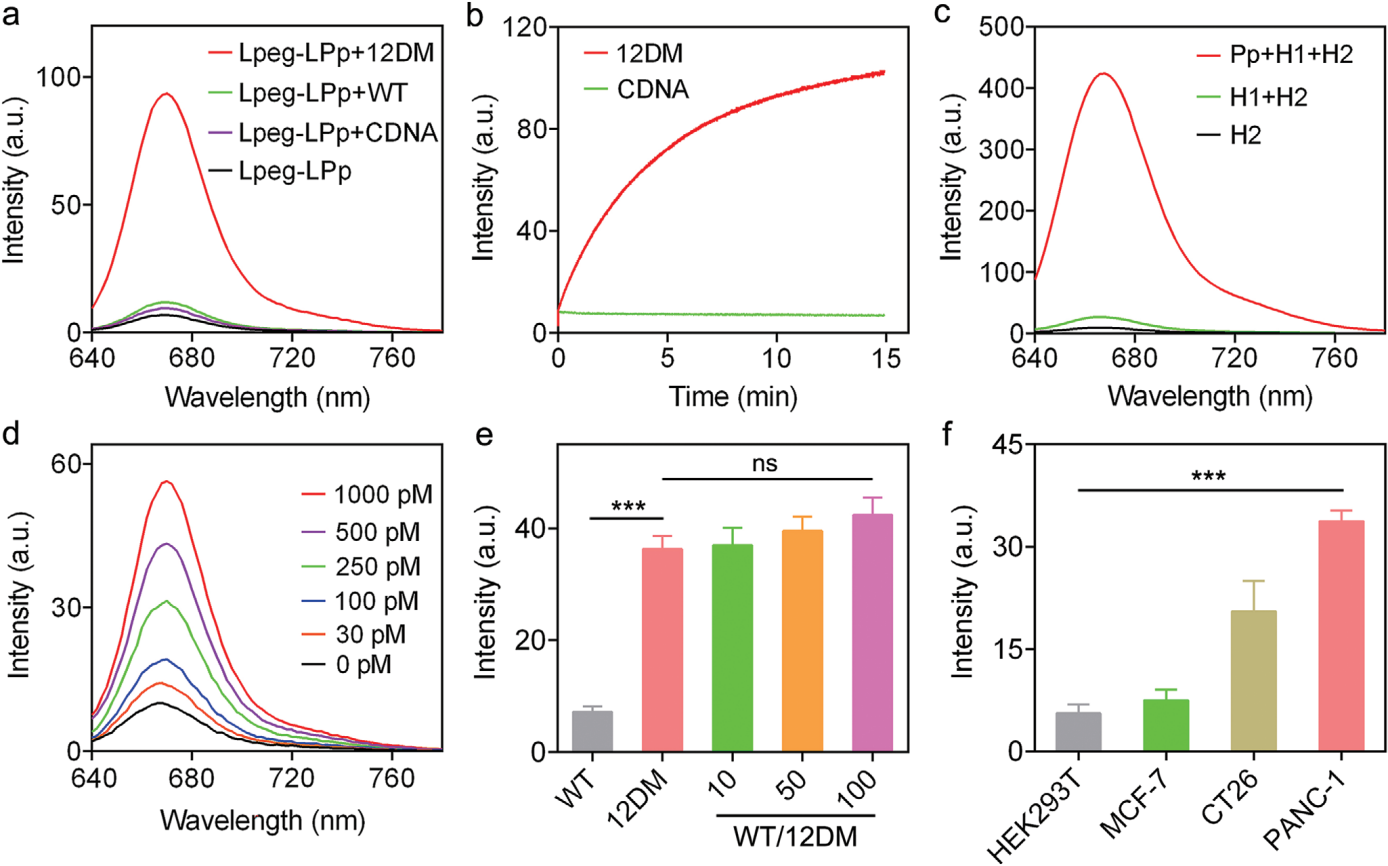
a) Fluorescence response of Lpeg‐LPp (LPp: 200 nm) to 12DM, WT and CDNA (30 nm). Excitation: 620 nm. b) Fluorescence kinetics of Lpeg‐LPp (LPp: 200 nm) to 12DM in comparison to CDNA (30 nm). Excitation: 620 nm. Emission: 670 nm. c) Fluorescence spectra of HCR with and without the addition of Pp (100 nm). Excitation: 620 nm. d) Fluorescence spectra of Lpeg‐LPp (LPp: 200 nm) in response to different concentrations of 12DM after signal amplification using HCR. Excitation: 620 nm. e) Fluorescence intensity of Lpeg‐LPp (LPp: 200 nm) challenged with 12DM (0.5 nm) in the presence of excess amount of WT strands. Excitation: 620 nm. Emission: 670 nm. f) Fluorescence response of Lpeg‐LPp (LPp: 200 nm) to different types of tumor cell DNA extracts. Excitation: 620 nm. Emission: 670 nm. Data are presented as means ± s.d. (n = 3). ns, not significant. ^***^ *P* < 0.001.

After that, we evaluated the sensing performance of Lpeg‐LPp to ctDNA mutation by choosing 12DM as a target. As shown in **Figures** **2**a and S3a,b (Supporting Information), dramatic fluorescence response (6.7‐fold to WT) could be detected in the presence of 12DM oligonucleotide in comparison to negligible fluorescence increment after WT or CDNA treatment and control nanoreporter Lpeg‐LPc, demonstrating the specificity of Lpeg‐LPp to 12DM. The selectivity of the γPNA to KRAS(G12D) mutation was further confirmed using a longer 12DM and WT oligonucleotides (80 bp) (Figure S3c, Supporting Information). It is reported that γPNA oligomer can invade double‐stranded DNA,^[^ ^44^ ^]^ we further investigated the fluorescence response of our nanoreporter to 12DM duplex (12DM^ds^). As shown in Figure S3d (Supporting Information), significant fluorescence increase was observed when 12DM^ds^ was added compared to background signal in WT^ds^ group. These results demonstrated that the Lpeg‐LPp could respond specifically to both single‐ and double‐stranded 12DM in buffer solution. The time‐dependent fluorescence response showed that the nanoreporter could recognize and respond to 12DM in several minutes (Figure 2b). We further designed the nanoreporter Lpeg‐LPs that target to KRAS(G12S) mutation and the fluorogenic assay results showed that significant fluorescence response could be observed in the presence of oligonucleotide containing G12S mutation compared to WT, demonstrating the generality of this system (Figure S3e, Supporting Information).

After confirming the specific recognition of Lpeg‐LPp to KRAS(G12D) mutation, we investigated the detection sensitivity by monitoring the dependence of fluorescence response on the concentration of 12DM. Concentration‐dependent fluorescence response was detected when the nanoreporter was treated with variable concentrations of 12DM as low as 1 nM (Figure S4, Supporting Information). To further improve the sensitivity and detection limit of Lpeg‐LPp, hybridization chain reaction (HCR), an efficient isothermal amplification strategy was employed for signal amplification.^[^ ^45^ ^]^ Gel electrophoresis results demonstrated that a lot of chain‐like duplex with long base‐pair strands were formed after incubating Pp with H1 and H2 hairpins (Figure S5a, Supporting Information). The fluorogenic assay showed that the fluorescence signal was dramatically amplified (≈30‐fold) when Pp was incubated with H1 and H2 (Figure 2c; Figure S5b, Supporting Information), demonstrating the feasibility of leveraging HCR for improving the sensitivity of Lpeg‐LPp in ctDNA mutation detection. We then evaluated the detection limit of the nanoreporter with the aid of the HCR amplification strategy by challenging Lpeg‐LPp with various concentrations of 12DM ranging from 30 pm to 1 nm. The fluorescence response increased with increasing concentrations of 12DM in comparison to the background fluorescence level in the control group (Figure 2d; Figure S6a,b, Supporting Information). The results demonstrated a good linear relationship between the fluorescence intensity and the logarithm value of target ctDNA concentration with a detection limit of 5.4 pm (Figure S6b, Supporting Information). To further investigate the selectivity of Lpeg‐LPp to 12DM, we challenged the nanoreporter with 12DM mixed with the excess amount of wild‐type KRAS strands at different ratios. Figure 2e showed that the response of Lpeg‐LPp to 12DM was not significantly affected in the presence of wild‐type strands, demonstrating good KRAS(12D) mutation specificity of the nanoreporter. Furthermore, we treated the Lpeg‐LPp with DNA extracts from HEK293T, MCF‐7, CT26, and PANC‐1 cell lines, among which PANC‐1 cells contained the most prevalent KRAS mutations.^[^ ^46^ ^]^ Dramatic fluorescence increase was detected after incubation with the extracts of CT26 and PANC‐1 cells containing KRAS mutations, whereas the response could be barely monitored after treatment with the extracts of HEK293T healthy cells and MCF‐7 breast cancer cells with almost no KRAS mutations (Figure 2f), which is consistent with the result of ddPCR (Figure S6c; Table S2, Supporting Information).^[^ ^8^ ^]^ Additionally, we also observed specific fluorescence response when the G12S‐specific nanoreporter Lpeg‐LPs were treated with lung cancer cell (A549) extracts compared to healthy cells due to the presence of G12S mutation in the tumor cells (Figure S6d, Supporting Information). Furthermore, the culture medium collected from PANC‐1 cells showed a significant fluorescence increase after Lpeg‐LPp treatment compared with the background signal of HEK293T group, demonstrating specific recognition of the nanoreporter to KRAS(G12D) mutation (Figure S6e, Supporting Information). Additionally, biostability analysis revealed that Lpeg‐LPp was stable (Figure S7a, Supporting Information) and the non‐specific release of Pp was negligible in biophysical environment (Figure S7b, Supporting Information). MTT assay results showed that the cytotoxicity of the nanoreporter was negligible (Figure S7c, Supporting Information). Collectively, these results demonstrated that the Lpeg‐LPp nanoreporter was successfully engineered for highly sensitive and specific KRAS(G12D) mutation detection.

### 2.2 In Vivo Clearance Pathway of the Nanoreporter

**Figure 3:**
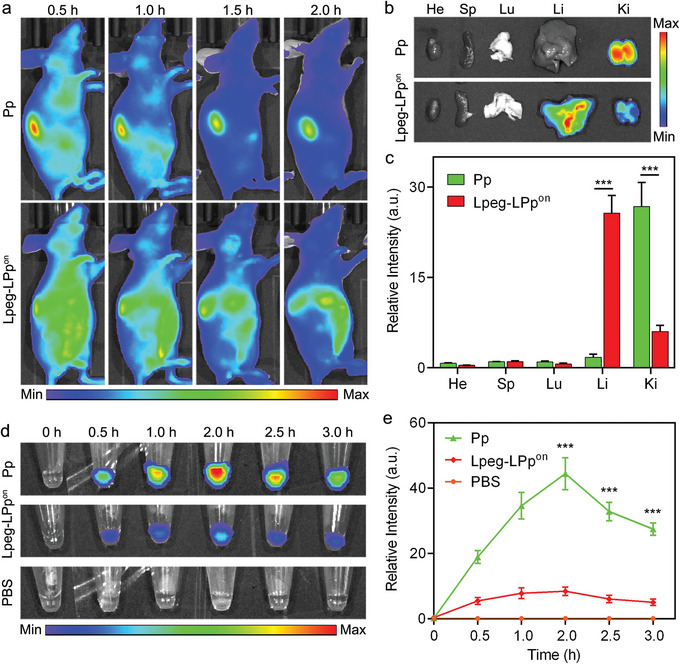
Biodistribution and clearance pathway of Lpeg‐LPp. a) Representative whole‐body fluorescence images of mice injected with Pp and Lpeg‐LPp^on^ (Pp: 100 nmol kg^−1^), respectively. Excitation: 640 nm. Emission: 670 nm. b) Fluorescence images of main organs (heart: He, spleen: Sp, lung: Lu, liver: Li, kidney: Ki) harvested from the mice in (a) at the end of the experiment. Excitation: 640 nm. Emission: 670 nm. c) Fluorescence signal quantification of main organs in (b). d) Fluorescence images of urine samples collected at indicated time points after the injection. Excitation: 640 nm. Emission: 670 nm. e) Fluorescence signal quantification of urine samples in (d). Data are presented as means ± s.d. (n = 3). ^***^ *P* < 0.001.

We next sought to investigate the clearance pathway of Lpeg‐LPp and Pp after intravenous injection. First, a blood elimination study showed that the half‐life of Pp was significantly increased after being ligated onto the 8‐arm PEG nanoparticle, and the introduction of albumin‐hitchhiking moiety further prolonged the blood circulation half‐life of Lpeg‐LPp (≈1.3 h) (Figure S8, Supporting Information). In vivo fluorescence imaging revealed that the always‐on (without BHQ3) nanoreporter Lpeg‐LPp^on^‐treated mice showed dramatic fluorescence increase throughout the body with the fluorescence in the liver gradually increased post‐injection. In contrast, the highest fluorescence signal was observed in the kidneys at 0.5 h post‐injection of Pp and LPp, and the signal gradually decreased in the following 2 h (**Figure** **3**a; Figure S9a, Supporting Information). To further confirm the biodistribution and clearance pathway, the fluorescence signal in excised organs and urine were detected. As shown in Figure 3b,c, the fluorescence in the liver of Lpeg‐LPp^on^‐treated mice is much stronger than that in the kidneys. On the contrary, the kidneys showed the highest fluorescence signal with minimal fluorescence in the liver of Pp and LPp groups (Figure 3b,c; Figure S9b,c, Supporting Information). Additionally, urine samples collected at different time points post‐injection of Pp and LPp showed much higher fluorescence signal than the nanoreporter group (Figure 3d,e; Figure S9d,e, Supporting Information), which is consistent with the in vivo fluorescence imaging results. These results revealed that the Pp and LPp were cleared through kidneys to urine, while Lpeg‐LPp was predominantly cleared through liver but not kidneys. This could be attributed to the larger size of Lpeg‐LPp than glomerulus pore (≈5 nm), which prevented the clearance of surface‐ligated LPp from the kidneys to urine before the dissociation triggered by KRAS(12D) mutation.^[^ ^47^ ^]^ Additionally, histological staining results of excised main organs revealed that both Pp and Lpeg‐LPp had high biocompatibility (Figure S10, Supporting Information).

### 2.3 Urinary Monitoring of Tumor and Prognosis using Lpeg‐LPp

**Figure 4:**
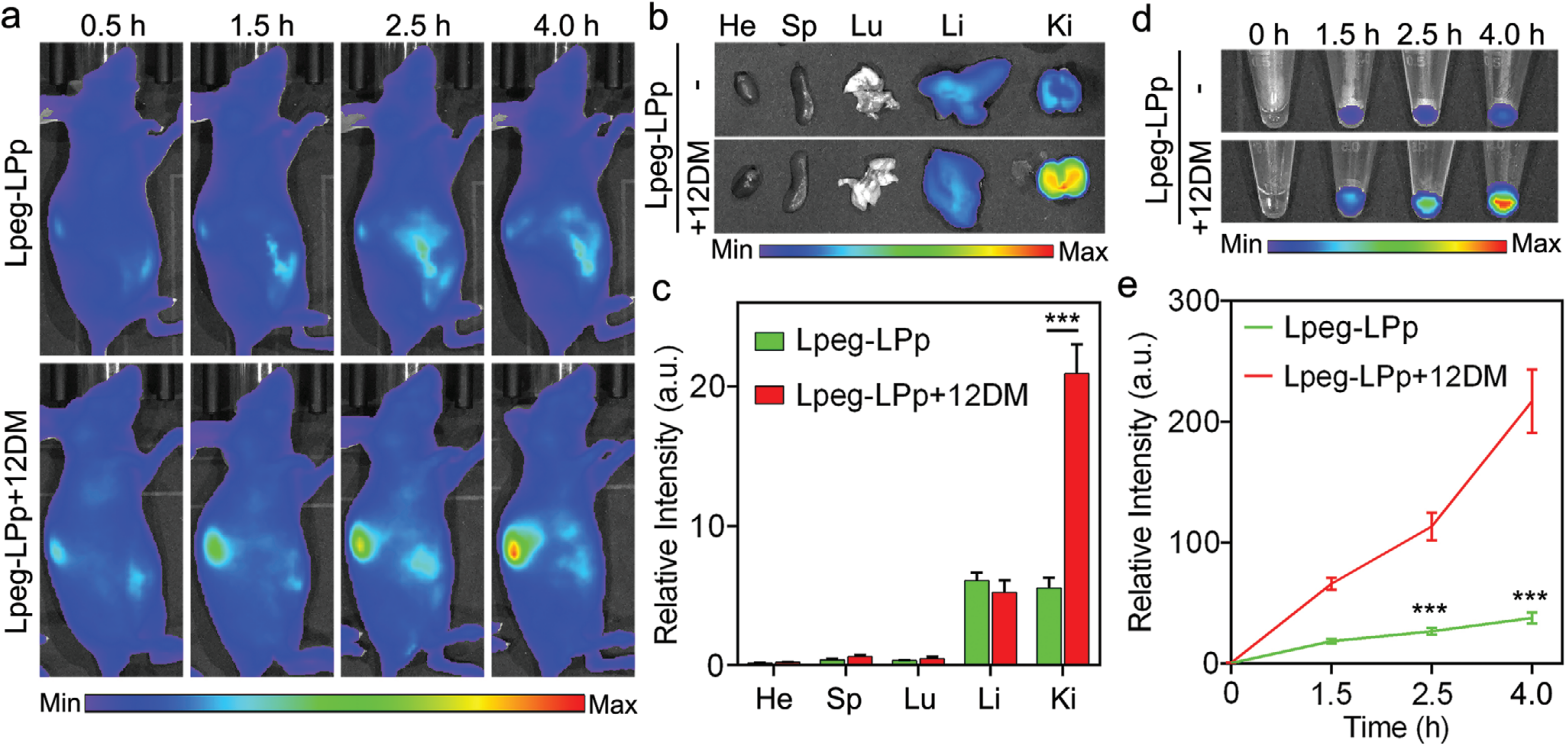
Fluorescence response of Lpeg‐LPp to supplemented 12DM oligonucleotide in peripheral blood. a) Representative whole‐body fluorescence images of mice injected with Lpeg‐LPp (Pp: 100 nmol kg^−1^) with and without supplementation of 12DM (5 nmol kg^−1^), respectively. Excitation: 640 nm. Emission: 670 nm. b) Fluorescence images of main organs (heart: He, spleen: Sp, lung: Lu, liver: Li, kidney: Ki) harvested from the mice in (a) at the end of experiment. Excitation: 640 nm. Emission: 670 nm. c) Fluorescence intensity quantification of main organs in (b). d) Fluorescence images of urine samples collected at indicated time points after the injection. Excitation: 640 nm. Emission: 670 nm. e) Signal intensity quantification of urine samples in (d). Data are presented as means ± s.d. (n = 3). ^***^ *P* < 0.001.

We first evaluated the response of Lpeg‐LPp to supplemented 12DM oligonucleotides in peripheral blood and the clearance pathway of Pp (Cy5‐labeled) after being detached from the nanoreporter. Two groups of healthy mice were intravenously administered with Lpeg‐LPp and Lpeg‐LPp+12DM, respectively. Whole‐body fluorescence imaging showed that the fluorescence signal at the kidney site gradually increased and reached a high level at 4 h post‐injection when 12DM was supplemented. In contrast, the mice injected with only the nanoreporter without 12DM supplementation showed minimal fluorescence increase in the kidneys (**Figure** **4**a). To further evaluate the biodistribution and clearance of Pp (Cy5‐labeled), urine samples were collected at indicated time points post‐injection, and the vital organs were harvested for *ex vivo* imaging. As shown in Figure 4b,c, the fluorescence intensity in the kidneys of Lpeg‐LPp+12DM group was ≈ 4.0‐ and 3.8‐fold stronger than those in the liver and the kidneys from Lpeg‐LPp group, respectively. Urinalysis demonstrated that the urine signal of the 12DM‐supplemented group gradually increased and showed ≈ 5.8‐fold higher intensity than that of the control group at 4 h post‐injection (Figure 4d,e), which correlated well with the *ex vivo* fluorescence imaging results. Collectively, these results revealed that Lpeg‐LPp could respond to KRAS(12D) mutation selectively in peripheral blood, and the clearance of dissociated Pp (Cy5‐labeled) from the kidneys to urine enabled the profiling of ctDNA mutation through urinalysis.

**Figure 5:**
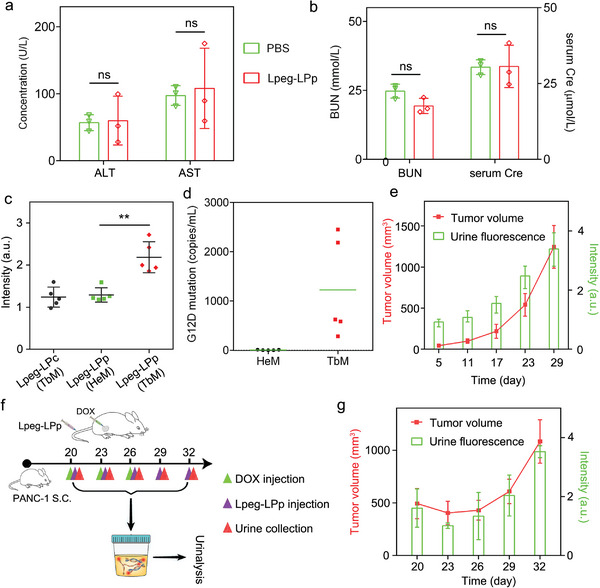
Urinary monitoring of tumor and prognosis using Lpeg‐LPp. Evaluation of a) liver and b) kidney function of mice by measuring the levels of ALT, AST, BUN, and serum Cre after Lpeg‐LPp treatment (Pp: 100 nmol kg^−1^) in comparison to PBS group. Data are presented as means ± s.d. (n = 3). c) Urinalysis of healthy and PDAC tumor‐bearing mice after Lpeg‐LPp injection. Excitation: 640 nm. Emission: 670 nm. Data are presented as means ± s.d. (n = 5). d) Quantitation of KRAS(G12D) mutation in the serum ctDNA using ddPCR. Data are presented as means ± s.d. (n = 5). e) Urinary monitoring of tumor progression using Lpeg‐LPp (Pp: 100 nmol kg^−1^). The urine fluorescence signal amplified by HCR gradually increased during tumor progression. Excitation: 640 nm. Emission: 670 nm. Data are presented as means ± s.d. (n = 3). f) Experimental design for urinary monitoring of tumor prognosis using Lpeg‐LPp. g) Urinary monitoring of tumor prognosis after intratumoral treatment of doxorubicin. The nanoreporter was intravenously injected and a urine sample was collected at indicated time points. The tumor size fluctuation after doxorubicin treatment and urine fluorescence intensity were quantified to show their correlation. Excitation: 640 nm. Emission: 670 nm. Data are presented as means ± s.d. (n = 3). ^**^ *P* < 0.01. ns, not significant.

Encouraged by the good performance of Lpeg‐LPp in the discrimination of supplemented 12DM oligonucleotide in peripheral blood, we sought to monitor the pancreatic ductal adenocarcinoma (PDAC) progression using the KRAS(12D) mutation‐responsive nanoreporter. The liver and kidney functions were first assessed after the mice were intravenously administered with the nanoreporter. The concentrations of alanine aminotransferase (ALT), aspartate aminotransferase (AST), blood urea nitrogen (BUN), and serum creatinine (Cre) showed negligible increment after the treatment in comparison to the PBS group (**Figure** **5**a,b), indicating the liver and kidney functions were intact after the treatment.

To explore whether Lpeg‐LPp could be employed for tumor diagnosis, a urinalysis of healthy mice and the PDAC‐bearing mice was conducted after the nanoreporter treatment. As shown in Figure 5c, much higher urine florescence signal (≈1.8‐fold) were detected for the tumor‐bearing mice (TbM) in comparison to healthy mice (HeM). While for control nanoreporter Lpeg‐LPc treated TbM, only background level of urine fluorescence could be detected. We further analyzed and quantified the ctDNA mutation in peripheral blood using ddPCR, a standard method for ctDNA mutation detection. The results revealed that the concentration of KRAS(G12D) mutation in the peripheral blood of PDAC tumor mice was much higher than that in healthy mice, which was consistent with the results obtained through urinalysis of γPNA after the Lpeg‐LPp treatment (Figure 5d; Figure S11, Supporting Information). After confirming the sensitivity of Lpeg‐LPp for tumor diagnosis, the potency of the nanoreporter for urinary monitoring of tumor progression was further evaluated. At indicated time points after intravenous injection of Lpeg‐LPp, the urine samples were collected for urinalysis (Figure S12a, Supporting Information). As shown in Figure 5e, both tumor size and urine fluorescence intensity concurrently increased during the time course of progression monitoring. The high correlation between urine signal and tumor size increment suggested the feasibility of leveraging Lpeg‐LPp for urinary recording of tumor progression. Ultimately, we explored whether the nanoreporter could be employed for tumor prognosis monitoring after chemotherapy. The tumor‐bearing mice were intratumorally administered with doxorubicin and further treated with Lpeg‐LPp intravenously at indicated time (Figure 5f,g). Urinalysis results indicated that the ctDNA level in peripheral blood decreased after the mice were treated with doxorubicin at the beginning of monitoring. However, it gradually increased from day 23 after second dosing, which was consistent with the results in previous literature reports.^[^ ^34^ ^]^ The tumor volume also demonstrated the similar fluctuations, which correlated well with that of ctDNA obtained through urinalysis. Additionally, no significant body weight changes were observed in all mice during the tumor progression and prognosis monitoring studies (Figure S12b,c, Supporting Information). Together, these results revealed that Lpeg‐LPp could be employed for urinary monitoring of tumor progression and prognosis through profiling and recording of ctDNA mutations in peripheral blood.

Although the nanoreporter showed high specificity for ctDNA mutation in peripheral blood, and urinalysis of cleared γPNA provided reliable information for tumor detection and prognosis evaluation, there are several limitations in the study. First, the sensitivity of the nanoreporter alone is limited, other signal amplification strategies such as HCR are needed to improve the sensitivity. Second, this study is limited to mouse tumor models, the detection sensitivity and biosafty profiles of our nanoreporter need to be carefully investigated on other models and patients. Thirdly, the application of our nanoreporter is limited by the tumor size due to its sensitivity. If the patients with very early‐stage cancer have the ctDNA concentration lower than the detection limit, the detection is challenging. Lastly, systemic administration of the nanoreporter is required before urinalysis, which may limit the applications.

## 3 Conclusion

In conclusion, we designed and constructed a DNA/γPNA hybrid nanoreporter that can specifically recognize and record KRAS(G12D) mutation in peripheral blood, allowing for urinary monitoring of tumor‐associated DNA mutation via γPNA urinalysis. The low tolerance of γPNA to single‐base mismatch in hybridization with DNA enabled highly selective recognition of ctDNA mutations. We verified that the γPNA strand could be detached from the nanoreporter selectively triggered by KRAS(G12D) mutation via toehold‐mediated strand displacement reaction. Urinalysis of the dissociated γPNA fragments cleared through kidneys to urine provide referable information of ctDNA mutations for tumor detection and prognosis evaluation. This work reveals a simple and feasible methodology to engineer DNA/γPNA nanoreporter for ctDNA mutations biosensing with high specificity in peripheral blood, which provides valuable information for tumor progression and prognosis evaluation via γPNA urinalysis. Considering the high ctDNA mutation selectivity and good biocompatibility of our nanoreporter, this study sheds light on an alternative strategy for ctDNA profiling and holds great potential for clinical translation applications.

## 4 Experimental Section

### Preparation of LPp and Lpeg‐LPp Nanoreporter

The LPp duplex was prepared by mixing BHQ3‐labeled linker DNA and Cy5‐labeled γPNA (10 µm, molar ratio 1:1) in HES buffer (120 mm NaCl, 5 mm MgCl_2_, 20 mm HEPES, pH 7.4). Then the mixture was incubated at 95 °C for 5 min and slowly cooled down to room temperature to obtain LPp duplex. The duplex was stored in the dark at 4 °C before using. To prepare the nanoreporter Lpeg‐LPp, the LPp duplex, and DSPE‐PEG‐DBCO were mixed with 8‐arm poly(ethylene glycol)‐N3 (40 kDa) at the ratio of 7:1:1 in HES buffer and incubated at room temperature for 2 h, followed by incubation at 4 °C overnight to obtain the Lpeg‐LPp. To evaluate the non‐specific Pp release from the surface of Lpeg‐LPp, the nanoreporter was diluted to 100 nm and incubated in HES buffer supplemented with 10% FBS for different time. Then the solution was ultrafiltered and the fluorecence of solution in the collection tube was measured using a microplate reader (ThermoFisher Scientific). Then the Pp concentration was calculated according to the linear relationship between Cy5 fluorescence intensity and concentration.

### Agarose gel Electrophoresis

The successful engineering of LPp and Lpeg‐LPp was confirmed through agarose gel electrophoresis. The prepared samples (linker DNA, Pp, LPp, Lpeg‐LPp) were mixed with 6 × DNA loading buffer (Invitrogen), followed by loading into the agarose gel matrix in 1 × TAE buffer and run at 120 V for 40 min. The gel FireRed images and fluorescence images were respectively acquired using a BioRad imaging system.

### KRAS^G12D^ ctDNA Mutation Sensing in Solution

The duplex and nanoreporters (LPp, Lpeg‐LPp, Lpeg‐LPc) were diluted to 200 nm if not indicated in HES buffer supplemented with different concentrations of DNA oligonucleotides (WT, 12DM, CDNA). The solutions were thoroughly mixed and incubated at 37 °C for 60 min. Then the fluorescence emission spectra were recorded (excitation wavelength: 620 nm, concentration: 200 nm, medium: HES buffer, temperature: 37 °C).

### γPNA‐Initiated HCR for Signal Amplification

The HCR‐based isothermal signal amplification strategy was employed to improve the detection sensitivity of Lpeg‐LPp. The designed H1 and H2 oligonucleotides (10 µm) in HES buffer were annealed by incubating at 95 °C for 5 min and slowly cool down to room temperature to obtain the H1 and H2 hairpins, respectively. The reaction products of Lpeg‐LPp mixed with target ctDNA were ultra‐filtrated to harvest the detached Pp or Pp/ctDNA hybrids. Then the harvested mixture was incubated at 60 °C for 30 min and further treated with DNase I (0.01 U µL^−1^) at 37 °C for 1 h, followed by thermal denaturation at 95 °C for 5 min to inactivate DNase. For HCR amplification analysis, the hairpins H1 and H2 (molar ratio 1:1) were added to the solution and incubated at 37 °C for 60 min, followed by fluorescence spectra collection.

### Cell Culture

HEK293T, MCF‐7, and PANC‐1 cell lines were cultured in DMEM medium supplemented with 10% FBS and 1% penicillin/streptomycin, and maintained in a humidified incubator containing 5% CO_2_ at 37 °C. A549 and CT26 cell lines were respectively cultured in F‐12K and RPMI‐1640 medium supplemented with 10% FBS and 1% penicillin/streptomycin, and maintained in a humidified incubator containing 5% CO_2_ at 37 °C.

### Biosensing of DNA Extracts or Secreted in Culture Medium

Nucleic acids in cells were extracted by DNA isolation Mini Kit (TIANGEN) according to the manufacturer's instructions. Then the isolated nucleic acids were treated with nanoreporter and the fluorescence signal were measured as the procedure described above. For the detection of target DNAs in the culture medium, the culture medium was collected when the cell confluence reached ≈90%. Then the medium was centrifuged at 10 000 g for 10 min at 4 °C. The supernatant was collected and further ultra‐filtrated to concentrate the DNAs. The collected nucleic acids were treated with the nanoreporter and the fluorescence spectra were collected.

### KRAS(G12D) Mutation Analysis using ddPCR

The analysis of KRAS(G12D) mutation in cell extracts or peripheral blood was performed using droplet digital PCR (ddPCR, Bio‐Rad QX200). The nucleic acids in blood were isolated using the QIAamp circulating nucleic acid kit (Qiagen). The ddPCR reaction mixture (20 µL) was assembled with ddPCR Supermixture for Probes (no dUTP), 1 µL of ctDNA template, 0.5 µL of probe, and 1.8 µL of forward‐ and reverse‐primers. The mixture was put on the plate of a droplet generator cartridge. Then 70 µL droplet generation oil was added into the well for droplets generation. The droplets were further transferred into a 96‐well PCR plate. After sealing and heating of the sample plate, the amplification can be started. The reaction started by template pre‐denature at 95 °C for 2 min, followed by 40 cycles of 30 s at 94 °C, 60 s at 60 °C, and then 10 min at 98 °C, lastly hold at 4 °C. The data was analyzed using a QuantaSoft after the amplification was finished.

### Cytotoxicity Assay

The cytotoxicity of Lpeg‐LPp nanoreporter was evaluated through MTT assay. HEK293T cells were seeded in a 96‐well plate and cultured for 24 h to reach 60% confluence. Then the cells were treated with culture medium supplemented with different concentrations of Lpeg‐LPp. After further cultured for 24 h, the medium was discarded and replaced with fresh cell culture medium supplemented with 10% MTT solution. After another 2 h incubation at 37 °C, the absorbance at 570 nm was measured using a microplate reader (ThermoFisher Scientific).

### In Vivo and Ex Vivo Fluorescence Imaging

All animal studies were performed according to the guidelines of the Institutional Animal Care and Use Committee (IACUC) of the Animal Experiment Center of Guangzhou Medical University (B2023‐073). To evaluate the clearance pathway of Pp and Lpeg‐LPp, the healthy mice without tumor were intravenously injected with 100 µL Pp, Lpeg‐LPp^on^, Lpeg‐LPp, and Lpeg‐LPp+12DM (5 nmol kg^−1^) at the Pp dosage of 100 nmol kg^−1^, respectively. At indicated time points after the dosing, the mice were anesthetized and fluorescence images were acquired using an IVIS Spectrum in vivo imaging system, followed by urine samples collection using a homemade box with 96‐well plates at the bottom. After the experiment, the mice were euthanized, the main organs were collected for ex vivo fluorescence imaging and sectioning for H&E staining analysis. To analyze the blood half‐life of Lpeg‐LPp, three mice in each group were intravenously injected with Pp, peg‐LPp^on^ (without DSPE module), Lpeg‐LPp^on^ at the Pp dosage of 100 nmol kg^−1^, then the blood samples were collected through the tail‐clip method. The fluorescence signal of urine and blood samples were respectively measured using the IVIS Spectrum in vivo imaging system (Excitation: 640 nm. Emission: 670 nm.). To investigate the biocompatibility of Lpeg‐LPp, the blood samples were collected through tail‐clip method 72 h after the nanoreporter and PBS injection, respectively. Then the concentration of serum ALT, AST, BUN, and creatinine were determined following the protocols of commercial kits.

### Urinalysis of Tumor and Prognosis Monitoring

For xenograft models implantation, the female BALB/c nude mice (6‐8 weeks) were subcutaneously injected with PANC‐1 cells (5 × 10^6^ cells/100 µL in PBS) on their right flanks. The tumor size was measured at the indicated time and volumes were calculated using the formula: V = length × width × width/2. When tumor volume reached ≈ 1000 mm^3^, the mice were randomly divided into 2 groups with 5 mice in each group. The two groups of tumor‐bearing mice were intravenously injected with Lpeg‐LPp and Lpeg‐LPc at the Pp or Ppc dosage of 100 nmol kg^−1^, respectively. The 5 healthy mice in the other control group were intravenously injected with Lpeg‐LPp. 2.5 h after each dosing, the urine samples were collected for further urinalysis. For tumor progression recording, tumor size was measured at the indicated time (day 5, 11, 17, 23, 29) after tumor inoculation (day 0). Meanwhile, the mice were intravenously injected with Lpeg‐LPp at the Pp dosage of 100 nmol kg^−1^, respectively. 2.5 h after each injection, the urine samples were collected for further analysis. For prognosis monitoring, the mice were intratumorally injected with doxorubicin (3 mg kg^−1^) at days 20, 23, and 26 when the tumor reached ≈ 500 mm^3^. Furthermore, the mice in each group were intravenously injected with Lpeg‐LPp at the Pp dosage of 100 nmol kg^−1^ 5 h after doxorubicin injection at days 20, 23, 26, 29, and 32, respectively. 2.5 h after each injection, the urine samples were collected using a custom housing with 96‐well plates at the bottom. For urinalysis, the urine samples were centrifuged at 2500 g for 10 min. The supernatant was collected and added to HES buffer (volume ratio 4:1). The solution was incubated at 60 °C for 30 min, followed by DNase I treatment at 37 °C for 1 h. After thermal denaturation at 95 °C for 5 min to inactivate DNase, the hairpins H1 and H2 (molar ratio 1:1) were added to the solution and further incubated at 37 °C for 90 min. The fluorescence intensity (excitation: 620 nm, emission: 670 nm) was measured using a microplate reader (ThermoFisher Scientific).

### Statistical Analysis

The sample size (n) for statistical analysis is included in figure legends. Data were processed and analyzed by one‐way analysis of variance (ANOVA) or unpaired Student's *t*‐test in GraphPad Prism software. ns, not significant, ^*^ *P* < 0.05, ^**^ *P* < 0.01 and ^***^ *P* < 0.001. All data in the manuscript were presented as means ± s.d.

## Conflict of Interest

The authors declare no conflict of interest.

## Supporting information

Supporting Information

## Data Availability

The data that support the findings of this study are available in the supplementary material of this article.
