# Supplementary material for: Engineering of a DNA/γPNA Hybrid Nanoreporter for ctDNA Mutation Detection via γPNA Urinalysis
Source: Adv Sci (Weinh). 2024 Jul 3;11(33):2310225. doi: 10.1002/advs.202310225 (PMC11434236; doi:10.1002/advs.202310225)
Supplement: Supplementary file 1 — Supporting Information [file ADVS-11-2310225-s001.docx]

Supporting information

**Engineering of a DNA/γPNA Hybrid Nanoreporter for ctDNA Mutation Detection via γPNA Urinalysis**

Zhichu Xiang, Jianhua Lu, Yang Ming, Weisheng Guo*, Xiaoyuan Chen*, and Weijian Sun*

Z. Xiang, J. Lu, W. Sun

Department of Gastrointestinal Surgery, the Second Affiliated Hospital and Yuying Children's Hospital of Wenzhou Medical University, Wenzhou 325027, China.
E-mail: fame198288@126.com

Z. Xiang, Y. Ming, X. Chen
Departments of Diagnostic Radiology, Surgery, Chemical and Biomolecular Engineering, and Biomedical Engineering, Yong Loo Lin School of Medicine and College of Design and Engineering, National University of Singapore, Singapore 119074, Singapore.

E-mail: chen.shawn@nus.edu.sg.

X. Chen
Clinical Imaging Research Centre, Centre for Translational Medicine, Yong Loo Lin School of Medicine, National University of Singapore, Singapore 117599, Singapore.

X. Chen
Nanomedicine Translational Research Program, Yong Loo Lin School of Medicine, National University of Singapore, Singapore 117597, Singapore.

X. Chen

Theranostics Center of Excellence (TCE), Yong Loo Lin School of Medicine, National University of Singapore, 11 Biopolis Way, Helios, Singapore 138667

X. Chen
Institute of Molecular and Cell Biology, Agency for Science, Technology, and Research (A*STAR), 61 Biopolis Drive, Proteos, Singapore 138673, Singapore.

W. Guo
Department of Minimally Invasive Interventional Radiology, the State Key Laboratory of Respiratory Disease, School of Biomedical Engineering & The Second Affiliated Hospital, Guangzhou Medical University, Guangzhou, 510260, China.

E-mail: tjuguoweisheng@126.com.

**Materials and methods**

**Materials.** All DNA oligonucleotides (Table S1) used in this study were synthesized and purified by Integrated DNA Technologies (IA, USA). The γPNA oligomers were synthesized and purified by PANAGENE (Daejeon, Korea) and used as supplied without further purification. 8-arm 40-kDa poly(ethylene glycol)-azido (8PEG-N3) and DSPE-PEG-DBCO (2kDa) were purchased from Ponsure Biotech Inc. DNase I for nonspecific DNA degradation was bought from ThermoFisher (MA, USA). Fetal bovine serum (FBS) was purchased from Gibco (CA, USA). The assay kits for ALT, AST, BUN and serum creatinine level evaluation were bought from Sigma-Aldrich. The mice used in this study were obtained from Charles River (Beijing, China).

**Instrumentations.** A FS5 Spectrofluorometer (Edinburgh, UK) was used to analyze the fluorescence spectra. The Milli-Q water used in this study was acquired from a Millipore ultrapure water system (Billerica, USA). Agarose gel electrophoresis images were acquired using BioRad imaging system (Biorad, USA). The absorbance at 570 nm and fluorescence emission at 670 nm (excitation 620 nm) were measured using a microplate reader (ThermoFisher Scientific). Both *ex vivo* and *in vivo* fluorescence images were acquired using an IVIS SPECTRUM *in vivo* imaging system (PerkinElmer, USA).

**Preparation of LPp and Lpeg-LPp nanoreporter.** The LPp duplex was prepared by mixing BHQ3-labeled linker DNA and Cy5-labeled γPNA (10 μM, molar ratio 1:1) in HES buffer (120 mM NaCl, 5 mM MgCl_2_, 20 mM HEPES, pH 7.4). Then the mixture was incubated at 95 ℃ for 5 min and slowly cooled down to room temperature to obtain LPp duplex. The duplex was stored in dark at 4 ℃ before using. To prepare the nanoreporter Lpeg-LPp, the LPp duplex and DSPE-PEG-DBCO were mixed with 8-arm poly(ethylene glycol)-N3 (40kDa) at the ratio of 7:1:1 in HES buffer and incubated at room temperature for 2 h, followed by incubation at 4 ℃ overnight to obtain the Lpeg-LPp.

**Agarose gel electrophoresis.** The successful engineering of LPp and Lpeg-LPp was confirmed through agarose gel electrophoresis. The prepared samples (linker DNA, Pp, LPp, Lpeg-LPp) were mixed with 6 × DNA loading buffer (Invitrogen), followed by loading into the agarose gel matrix in 1 × TAE buffer and run at 120 V for 40 min. The gel FireRed images and fluorescence images were respectively acquired using a BioRad imaging system.

***KRAS^G12D^* ctDNA mutation sensing in solution.** The duplex and nanoreporters (LPp, Lpeg-LPp, Lpeg-LPc) were diluted to 200 nM if not indicated in HES buffer supplemented with different concentrations of DNA oligonucleotides (WT, 12DM, CDNA). The solutions were thoroughly mixed and incubated at 37 ℃ for 60 min. Then the fluorescence emission spectra were recorded with the excitation wavelength at 620 nm.

**γPNA-initiated HCR for signal amplification.** The HCR-based isothermal signal amplification strategy was employed to improve the detection sensitivity of Lpeg-LPp. The designed H1 and H2 oligonucleotides (10 μM) in HES buffer were annealed by incubating at 95 °C for 5 min and slowly cool down to room temperature to obtain the H1 and H2 hairpins, respectively. The reaction products of Lpeg-LPp mixed with target ctDNA were ultra-filtrated to harvest the detached Pp or Pp/ctDNA hybrids. Then the harvested mixture was incubated at 60 °C for 30 min and further treated with DNase Ⅰ (0.01 U/μL) at 37 °C for 1 h, followed by thermal denaturation at 95 °C for 5 min to inactivate DNase. For HCR amplification analysis, the hairpins H1 and H2 (molar ratio 1:1) were added to the solution and incubated at 37 °C for 60 min, followed by fluorescence spectra collection.

**Cell culture.** HEK293T and PANC-1 cell lines were cultured in DMEM medium supplemented with 10% FBS and 1% penicillin/streptomycin, and maintained in a humidified incubator containing 5% CO_2_ at 37 ℃.

**Biosensing of DNA extracts or secreted in culture medium.** Nucleic acids in cells were extracted by DNA isolation Mini Kit (TIANGEN) according to the manufacture instructions. Then the isolated nucleic acids were treated with nanoreporter and the fluorescence signal were measured as the procedure described above. For detection of target DNAs in culture medium, the culture medium was collected when the cell confluence reached about 90%. Then the medium was centrifuged at 10000 g for 10 min at 4 °C. The supernatant was collected and further ultra-filtrated to concentrate the DNAs. The collected nucleic acids were treated with the nanoreporter and the fluorescence spectra were collected.

**KRAS(G12D) mutation analysis using ddPCR.** The analysis of KRAS(G12D) mutation in cell extracts or peripheral blood was performed using droplet digital PCR (ddPCR, Bio-Rad QX200). The nucleic acids in blood were isolated using the QIAamp circulating nucleic acid kit (Qiagen). The ddPCR reaction mixture (20 μL) was assembled with ddPCR Supermixture for Probes (no dUTP), 1 μL of ctDNA template, 0.5 μL of probe, and 1.8 μL of forward- and reverse-primers. The mixture was put on the plate of a droplet generator cartridge. Then 70 μL droplet generation oil was added into the well for droplets generation. The droplets were further transferred into a 96-well PCR plate. After sealing and heating of the sample plate, the amplification can be started. The reaction started by template pre-denature at 95 ℃ for 2 min, followed by 40 cycles of 30 s at 94 ℃, 60 s at 60 ℃, and then 10 min at 98 ℃, lastly hold at 4 ℃. The data was analyzed using a QuantaSoft after the amplification was finished.

**Cytotoxicity assay.** The cytotoxicity of Lpeg-LPp nanoreporter was evaluated through MTT assay. HEK293T cells were seeded in a 96-well plate and cultured for 24 h to reach 60% confluence. Then the cells were treated with culture medium supplemented with different concentrations of Lpeg-LPp. After further cultured for 24h, the medium was discarded and replaced with fresh cell culture medium supplemented with 10% MTT solution. After another 2 h incubation at 37 ℃, the absorbance at 570 nm was measured using a microplate reader (ThermoFisher Scientific).

***In vivo* and *ex vivo* fluorescence imaging.** All animal studies were performed according to the guidelines of Institutional Animal Care and Use Committee (IACUC) of Animal Experiment Center of Guangzhou Medical University (B2023-073). To evaluate the clearance pathway of Pp and Lpeg-LPp, the healthy mice without tumor were intravenously injected with 100 μL Pp, Lpeg-LPp^on^, Lpeg-LPp and Lpeg-LPp+12DM (5 nmol/kg) at the Pp dosage of 100 nmol/kg, respectively. At indicated time points after the dosing, the mice were anesthetized and fluorescence images were acquired using an IVIS Spectrum *in vivo* imaging system (Excitation: 640 nm. Emission: 670 nm), followed by urine samples collection using a homemade box with 96-well plates at the bottom. After the experiment, the mice were euthanized, the main organs were collected for *ex vivo* fluorescence imaging and sectioning for H&E staining analysis. To analyze the blood half-life of Lpeg-LPp, three mice in each group were intravenously injected with Pp, peg-LPp^on^ (without DSPE module), Lpeg-LPp^on^ at the Pp dosage of 100 nmol/kg, then the blood samples were collected through tail-clip method. The fluorescence signal of urine and blood samples were respectively measured using the IVIS Spectrum *in vivo* imaging system. To investigate the biocompatibility of Lpeg-LPp, the blood samples were collected through tail-clip method 72 h after the nanoreporter and PBS injection, respectively. Then the concentration of serum ALT, AST, BUN and creatinine were determined following the protocols of commercial kits.

**Urinalysis of tumor and prognosis monitoring.** For xenograft models implantation, the female BALB/c nude mice (6-8 weeks) were subcutaneously injected with PANC-1 cells (5 × 10^6^ cells/100 μL in PBS) on their right flanks. The tumor size was measured at indicated time and volumes were calculated using the formula: V = length × width × width/2. When tumor volume reached about 1000 mm^3^, the mice were randomly divided into 2 groups with 5 mice in each group. The two groups of tumor-bearing mice were intravenously injected with Lpeg-LPp and Lpeg-LPc at the Pp or Ppc dosage of 100 nmol/kg, respectively. The 5 healthy mice in the other control group were intravenously injected with Lpeg-LPp. 2.5 h after each dosing, the urine samples were collected for further urinalysis. For tumor progression recording, tumor size was measured at indicated time (day 5, 11, 17, 23, 29) after tumor inoculation (day 0). Meanwhile, the mice were intravenously injected with Lpeg-LPp at the Pp dosage of 100 nmol/kg, respectively. 2.5 h after each injection, the urine samples were collected for further analysis. For prognosis monitoring, the mice were intratumorally injected with doxorubicin (3 mg/kg) at day 20, 23 and 26 when the tumor reached about 500 mm^3^. Furthermore, the mice in each group were intravenously injected with Lpeg-LPp at the Pp dosage of 100 nmol/kg 5 h after doxorubicin injection at day 20, 23, 26, 29 and 32, respectively. 2.5 h after each injection, the urine samples were collected using a custom housing with 96-well plates at the bottom. For urinalysis, the urine samples were centrifuged at 2500 g for 10 min. The supernatant was collected and added to HES buffer (volume ratio 4:1). The solution was incubated at 60 °C for 30 min, followed by DNase Ⅰ treatment at 37 °C for 1 h. After thermal denaturation at 95 °C for 5 min to inactivate DNase, the hairpins H1 and H2 (molar ratio 1:1) were added to the solution and further incubated at 37 °C for 90 min. The fluorescence intensity was measured using a microplate reader (ThermoFisher Scientific).

**Statistical Analysis.** The sample size (n) for statistical analysis is included in figure legends. Data were processed and analyzed by one-way analysis of variance (ANOVA) or unpaired Student’s t test in GraphPad Prism software. ns, not significant, *P < 0.05, **P < 0.01 and ***P < 0.001. All data in the manuscript were presented as means ± s.d.

Table S1. The sequences of DNA and PNA used in this study.

| **Name** | **Sequence (5’-3’ or N-C terminus)** |
| --- | --- |
| Lin | DBCO-peg6-GTTGG^#^AG^#^CT^#^G |
| LinQ | DBCO-peg6- GTTGG^#^AG^#^CT^#^G-BHQ3 |
| Pp | K-cat*cagctccaac-K  (the capital letter denotes amino acid, small letter denotes PNA base) |
| Pp (Cy5 labelled) | Cy5- K-cat*cagctccaac-K  (the capital letter denotes amino acid, small letter denotes PNA base) |
| LinQ1 (for G12S) | DBCO-peg6- GTTGG^#^AG^#^CT^#^A-BHQ3 |
| Ps (Cy5 labelled, for G12S) | Cy5- K-cac*tagctccaac-K  (the capital letter denotes amino acid, small letter denotes PNA base) |
| Ppc | Cy5- K-ttc*cagctccaac-K  (the capital letter denotes amino acid, small letter denotes PNA base) |
| 12DM | GTTGGAGCTGATGGCGTAG |
| 12SM | GTTGGAGCTAGTGGCGTAG |
| WT | GTTGGAGCTGGTGGCGTAG |
| 12DM^L^ | ATAGTCACATTTTCATTATTTTTATTATAAGGCCTGCTGAAAATGACTGAATATAAACTTGTGGTAGTTGGAGCTGATGG |
| WT^L^ | ATAGTCACATTTTCATTATTTTTATTATAAGGCCTGCTGAAAATGACTGAATATAAACTTGTGGTAGTTGGAGCTGGTGG |
| CDNA | TCCATGACGTTCCTGACGTT |
| Com | CTACGCCACCAGCTCCAAC |
| H1 | GTTGGAGCTGATGTCTCTCACATCAGCT |
| H2 | ACA/iBHQ2dT/CAGCTCCAACAGCTGA/iCy5dT/GTGAGAG |
| F-primer | TGCTGAAAATGACTGAATATAAACTTGTG |
| R-primer | AGCTGTATCGTCAAGGCACTCTT |
| G12D Probe (ddPCR) | FAM-TACGCCATCAGCTC-MGB |
| WT Probe (ddPCR) | VIC-ACGCCACCAGCTC-MGB |

^#^denotes phosphorothioate (PS) bond, *denotes gamma alanine-substituted PNA.


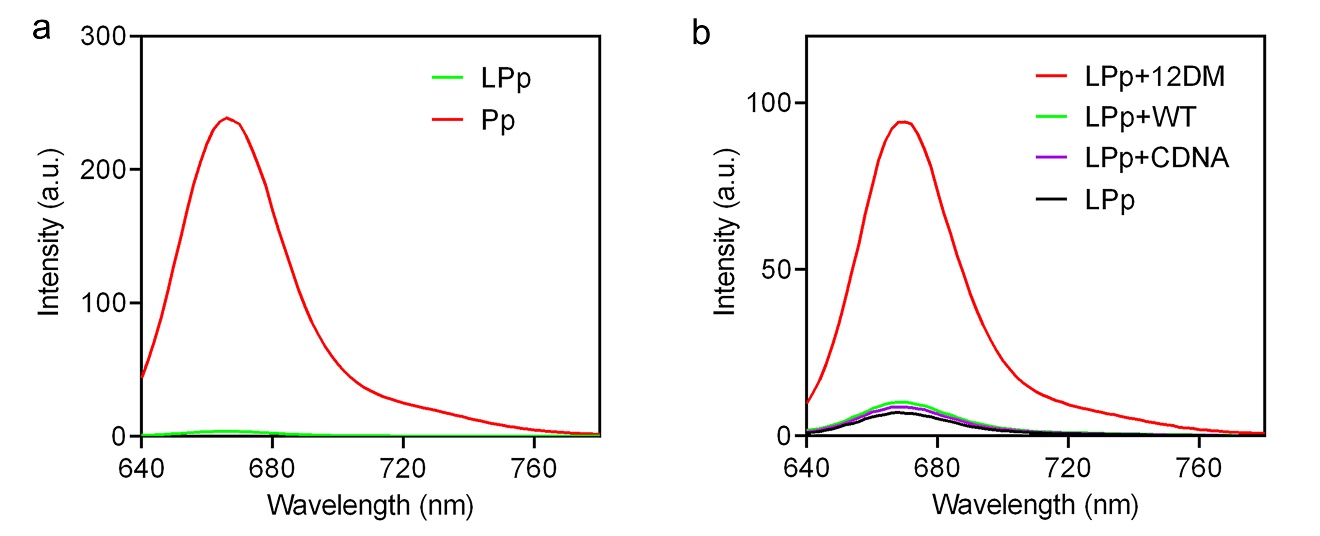


**Figure S1.** a) Fluorescence spectra of Pp (200 nM) before and after hybridized with LinQ. Excitation: 620 nm. b) Fluorescence response of LPp to 12DM, WT and CDNA (30 nM). Excitation: 620 nm.


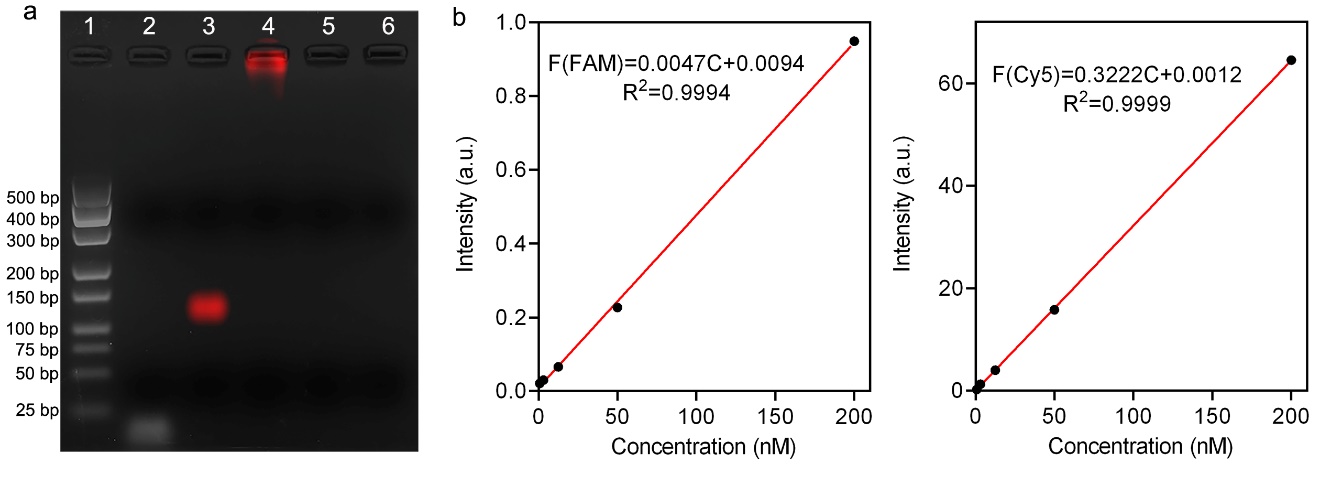


**Figure S2.** a) Agarose gel analysis of successful engineering of Lpeg-LPp nanoreporter. Lane 1: DNA ladder (25 bp); Lane 2: Lin (10 bp, single-stranded); Lane 3: LPp^on^ (without BHQ3, DNA/γPNA hybrid, DNA: 10 bp, γPNA: 13 bp); Lane 4: Lpeg-LPp^on^ (LPp^on^ ligated on PEG nanoparticle); Lane 5: LPp (with BHQ3, DNA/γPNA hybrid, DNA: 10 bp, γPNA: 13 bp); Lane 6: Lpeg-LPp (LPp ligated on PEG nanoparticle). b) The calibration curve showed linear relationship between fluorescence intensity and the concentration of FAM (Excitation: 490 nm. Emission: 520 nm) and Cy5 (Excitation: 620 nm. Emission: 670 nm), respectively.


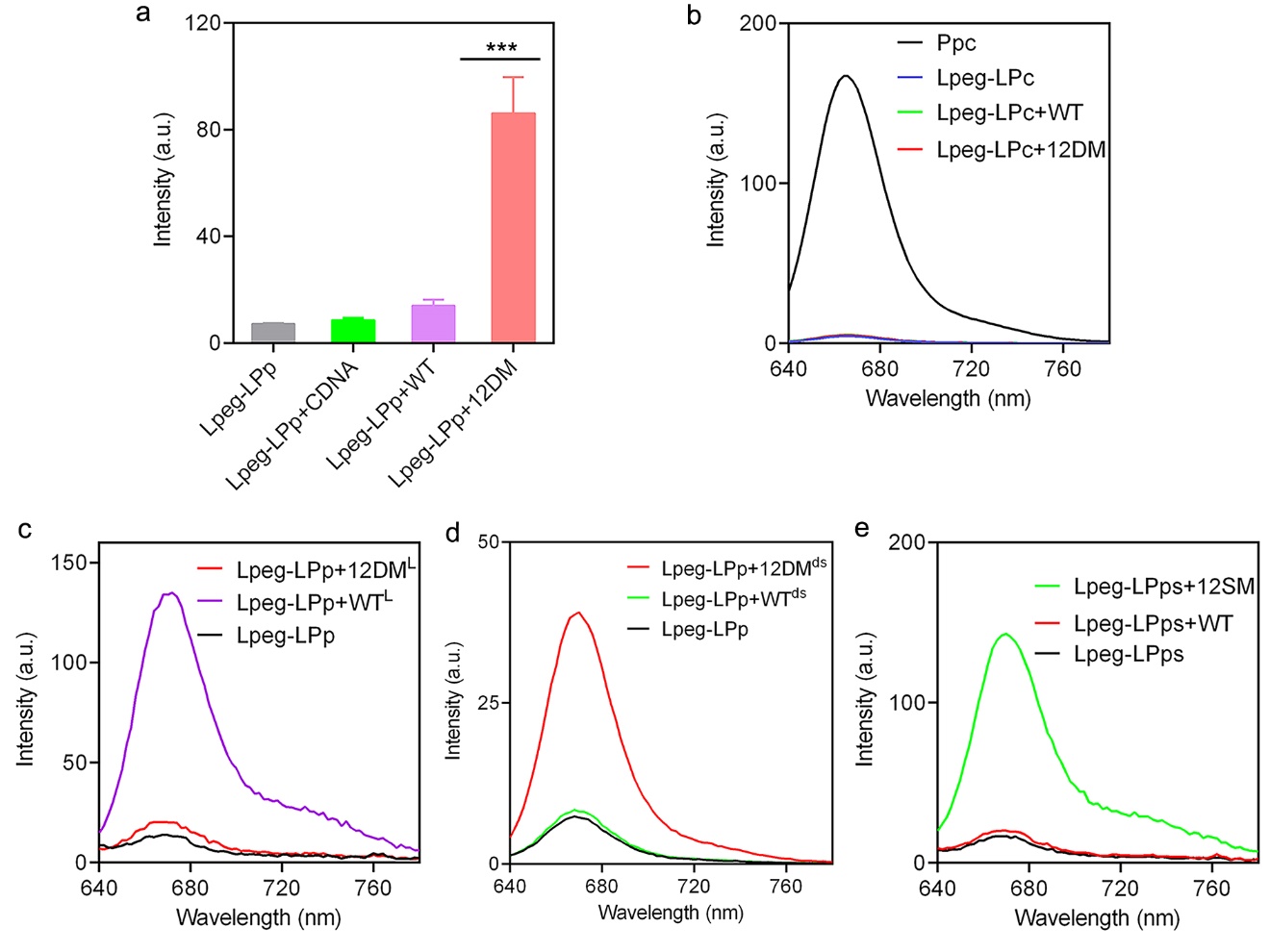


**Figure S3.** a) Fluorescence intensity of Lpeg-LPp (Pp: 200 nM) before and after respectively treated with 12DM, WT and CDNA (30 nM). Excitation: 620 nm. Emission: 670 nm b) Fluorescence response of Lpeg-LPc (Pc: 200 nM) to 12DM and WT in comparison to Ppc oligomer. Excitation: 620 nm. c) Fluorescence response of Lpeg-LPp (Pp: 200 nM) to longer 12DM^L^ and WT^L^ (80 bp). Excitation: 620 nm. d) Fluorescence response of Lpeg-LPp (Pp: 200 nM) to double-stranded 12DM and WT (30 nM). Excitation: 620 nm. e) Fluorescence response of G12S-specific nanoreporter Lpeg-LPs (Pps: 200 nM) to 30 nM KRAS(G12S) mutation and WT. Excitation: 620 nm. Data are presented as means ± s.d. (n = 3).


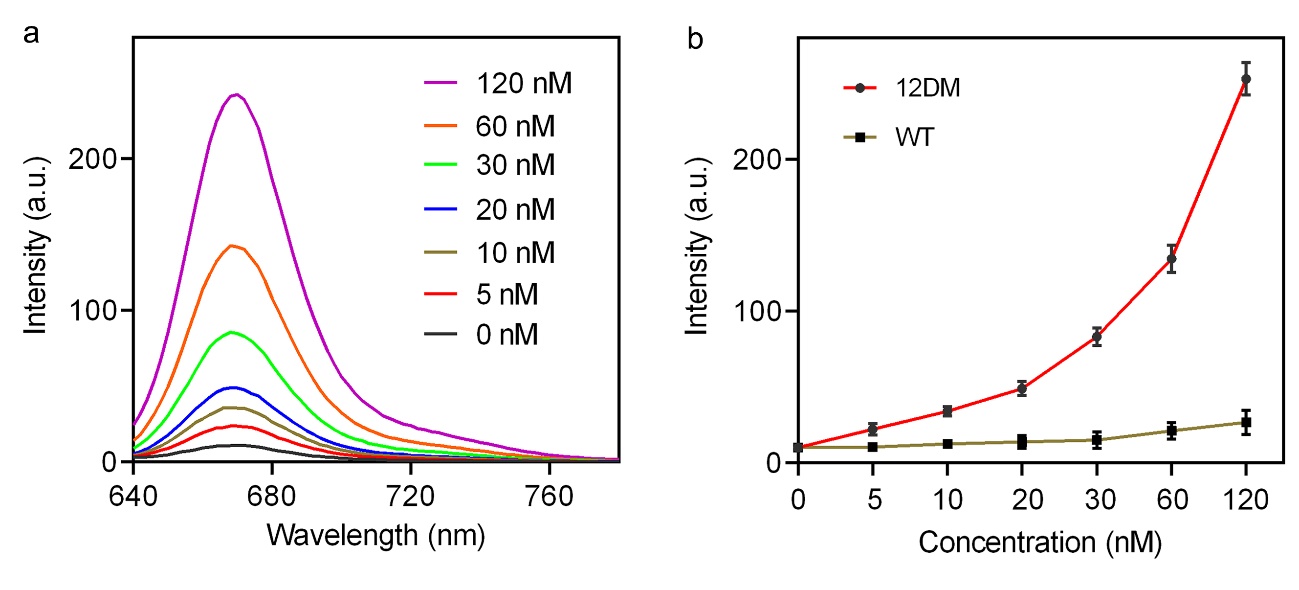


**Figure S4.** a) Fluorescence spectra of Lpeg-LPp (Pp: 200 nM) responding to different concentrations of 12DM. Excitation: 620 nm. b) Fluorescence intensity of Lpeg-LPp responding to different concentrations of 12DM and WT, respectively. Excitation: 620 nm. Emission: 670 nm. Data are presented as means ± s.d. (n = 3).


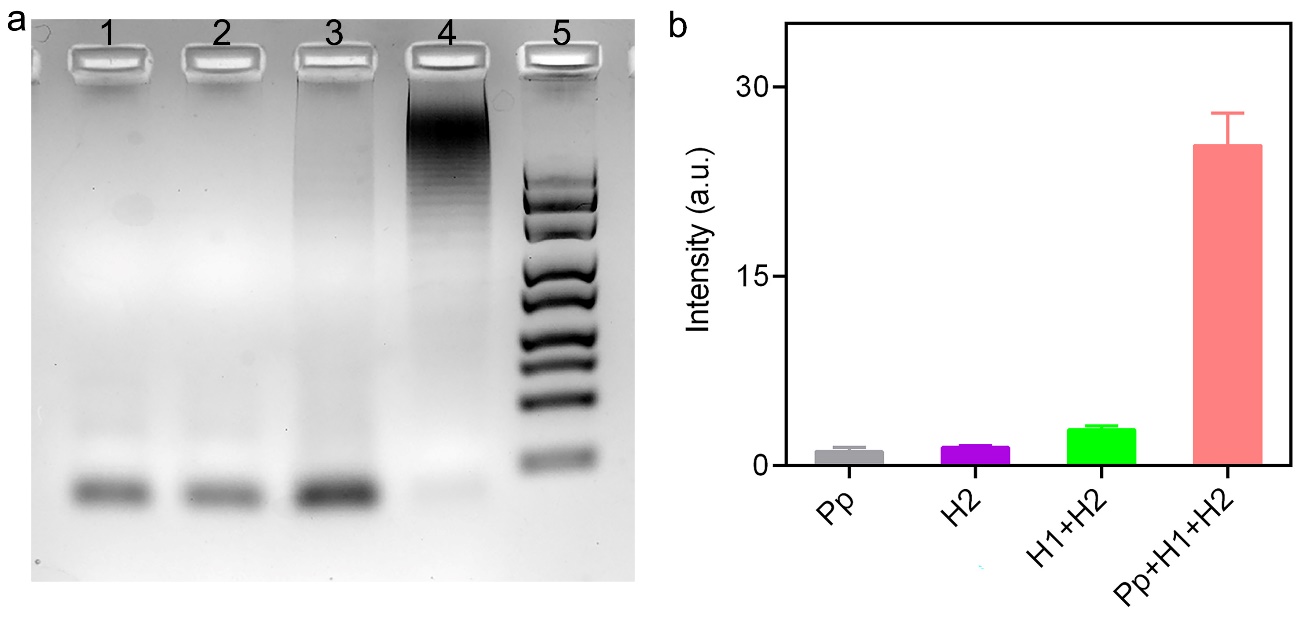


**Figure S5.** a) Agarose gel analysis of HCR with and without addition of Pp (100 nM). Lane 1: H1 (500 nM); Lane 2: H2 (500 nM); Lane 3: H1+H2; Lane 4: H1 + H2 + Pp; Lane 5: DNA ladder (25 bp). b) Fluorescence intensity of Pp before and after signal amplification using HCR. Excitation: 620 nm. Emission: 670 nm. Data are presented as means ± s.d. (n = 3).


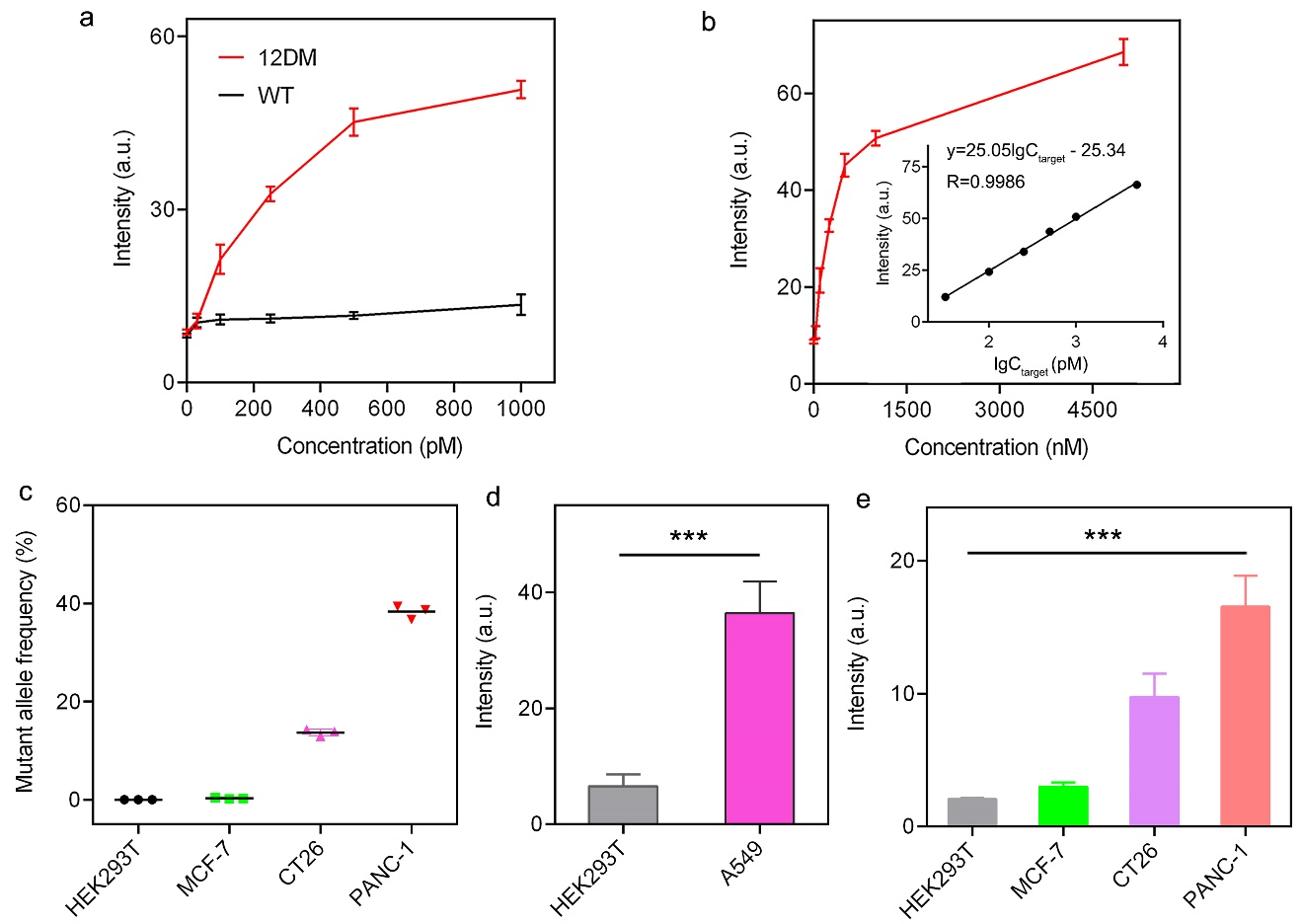


**Figure S6.** a) Fluorescence response of Lpeg-LPp (Pp: 200 nM) to different concentrations of 12DM and WT with the aid of HCR. Excitation: 620 nm. Emission: 670 nm. b) Linear relationship between fluorescence intensity and logarithm of target ctDNA with HCR-assisted amplification. Excitation: 620 nm. Emission: 670 nm. c) Quantitative analysis of KRAS(G12D) mutation in different cell extracts using ddPCR. d) Fluorescence intensity of G12S-specific nanoreporter Lpeg-LPs in response to KRAS(G12S) mutation in lung cancer cell (A549) extracts compared to healthy cells. Excitation: 620 nm. Emission: 670 nm. e) Fluorescence response of Lpeg-LPp to target DNA secreted by different cells in culture medium. Excitation: 620 nm. Emission: 670 nm. Data are presented as means ± s.d. (n = 3). ***P < 0.001.

Table S2. KRAS(G12D) mutation analysis of different cell extracts by ddPCR.

| **Cell** | **group** | | **Mutation（copies/µl）** | | **Wild type（copies/µl）** | | **Mutation frequency (%)** |
| --- | --- | --- | --- | --- | --- | --- | --- |
| HEK293T | 1 | 0 | | 142200 | | 0 | |
|  | 2 | 0 | | 142800 | | 0 | |
|  | 3 | 0 | | 137400 | | 0 | |
|  | 1 | 48 | | 16360 | | 0.29 | |
| MCF7 | 2 | 28 | | 16160 | | 0.17 | |
|  | 3 | 76 | | 17440 | | 0.43 | |
|  | 1 | 1060 | | 7120 | | 12.96 | |
| CT26 | 2 | 1159 | | 6960 | | 14.28 | |
|  | 3 | 1028 | | 6380 | | 13.88 | |
|  | 1 | 118000 | | 186800 | | 38.71 | |
| PANC-1 | 2 | 117600 | | 202400 | | 36.75 | |
|  | 3 | 126800 | | 194800 | | 39.43 | |


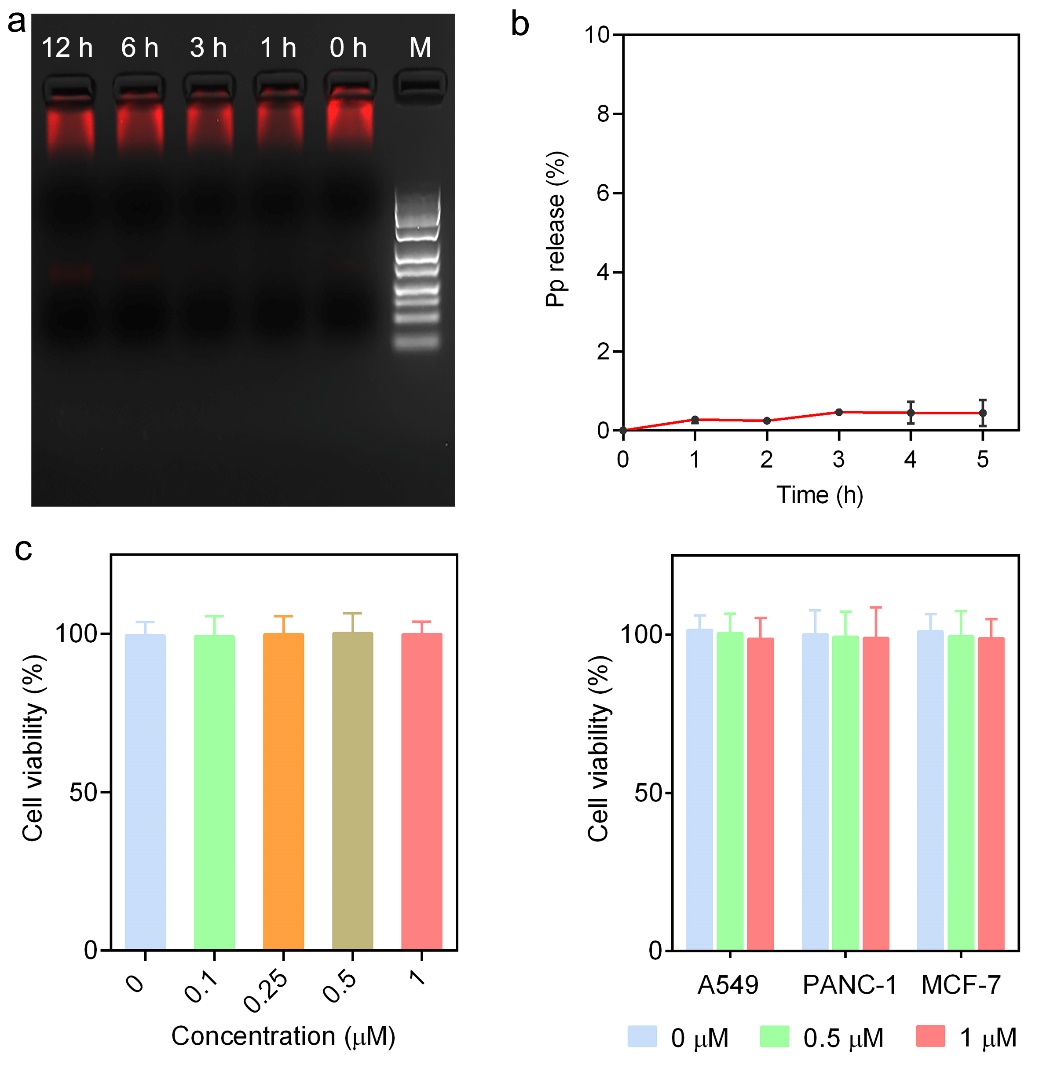


**Figure S7.** a) Agarose gel analysis of Lpeg-LPp (without quencher) stability in cell culture medium supplemented with 10% fetal bovine serum. b) non-specific Pp release from surface of the nanoraporter in buffer solution. Excitation: 620 nm. Emission: 670 nm. c) Cell viability of HEK293T (left), A549, PANC-1 and MCF-7 (right) cells after treatment with indicated concentrations of Lpeg-LPp. Data are presented as means ± s.d. (n = 3).


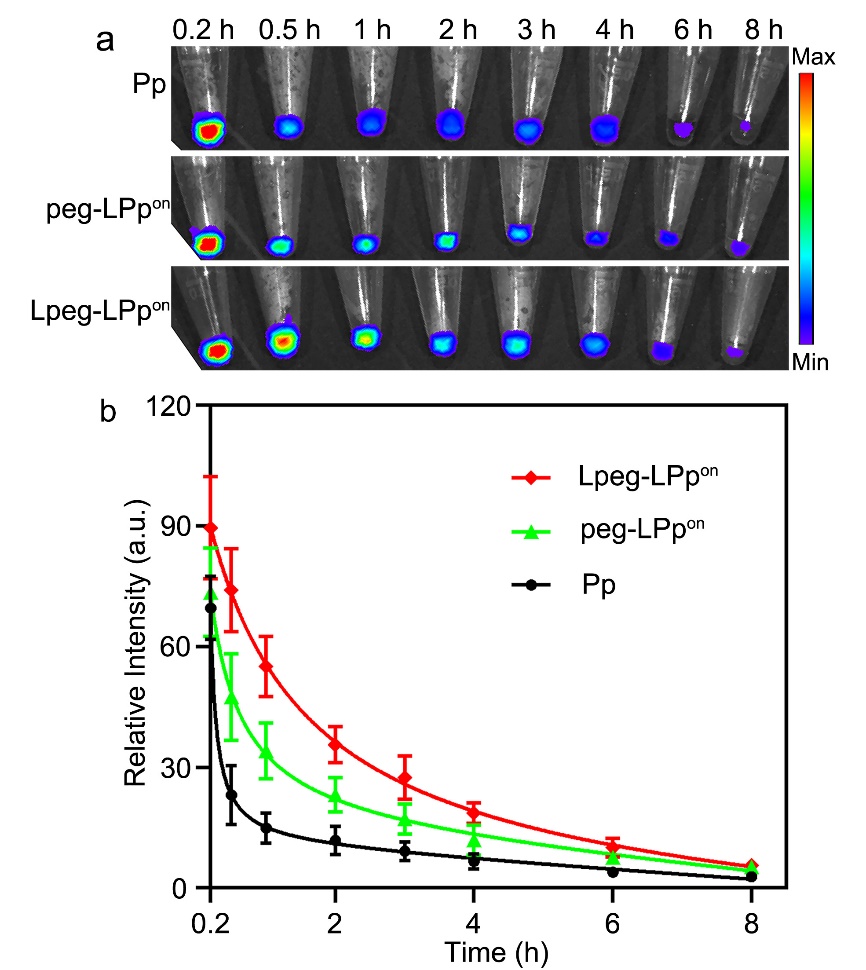


**Figure S8.** Blood circulation half-life evaluation of nanoreporter Lpeg-LPp. a) Representative fluorescence images of blood samples collected at indicated time points after injection. Excitation: 640 nm. Emission: 670 nm. b) Quantification and fitting of blood fluorescence intensity in (a). Data are presented as means ± s.d. (n = 3).


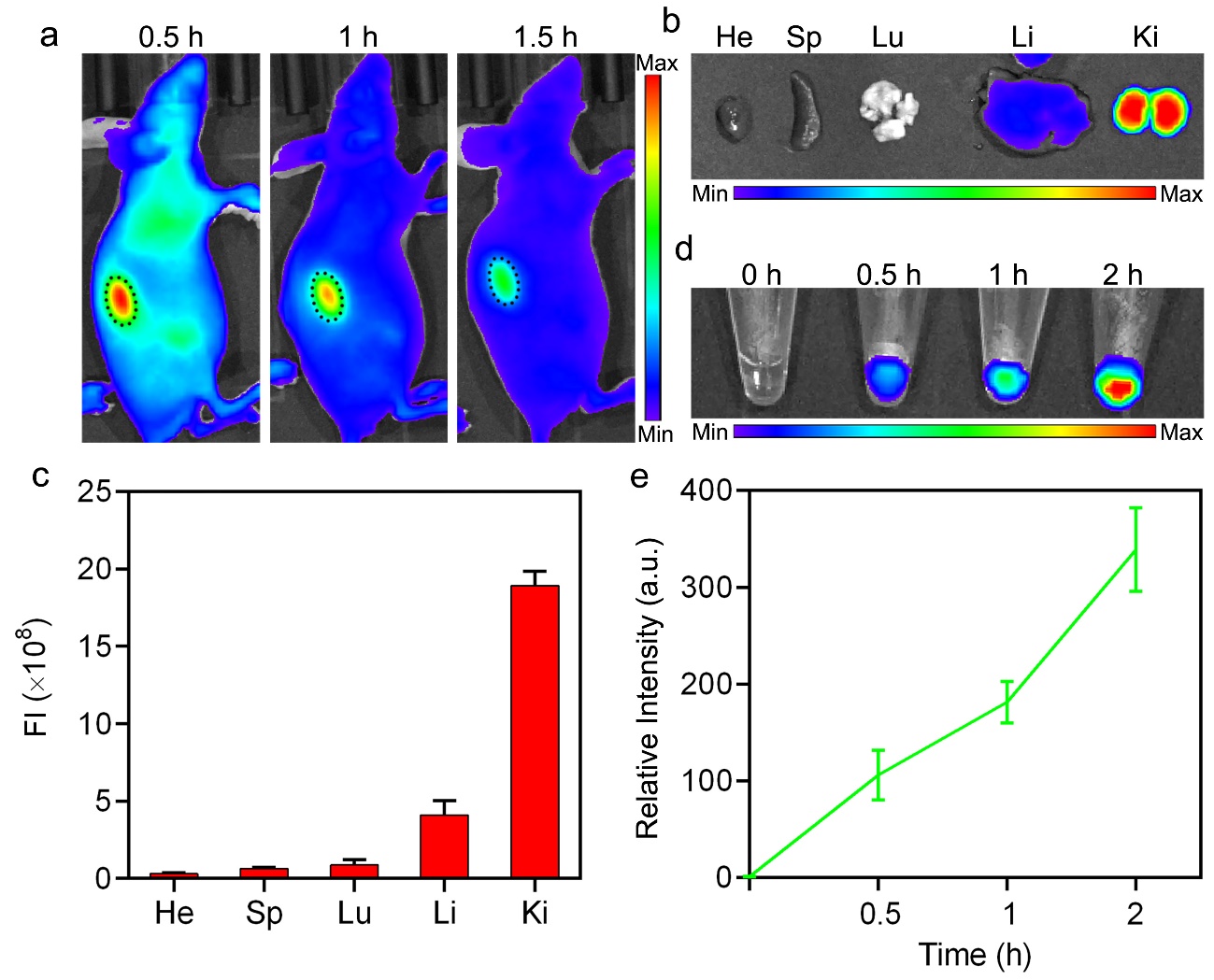


**Figure S9.** Biodistribution and clearance pathway of LPp. a) Representative fluorescence images of mice injected intravenously with LPp (Pp: 100 nmol/kg). Excitation: 640 nm. Emission: 670 nm. b) Fluorescence images of excised main organs (heart: He, spleen: Sp, lung: Lu, liver: Li, kidney: Ki) harvested at 2 h after the injection. Excitation: 640 nm. Emission: 670 nm. c) Fluorescence signal quantification of the main organs in (b). d) Fluorescence images of urine samples collected at indicated time points after the injection. Excitation: 640 nm. Emission: 670 nm. e) Relative fluorescence intensity of urine samples in (d). Data are presented as means ± s.d. (n = 3).


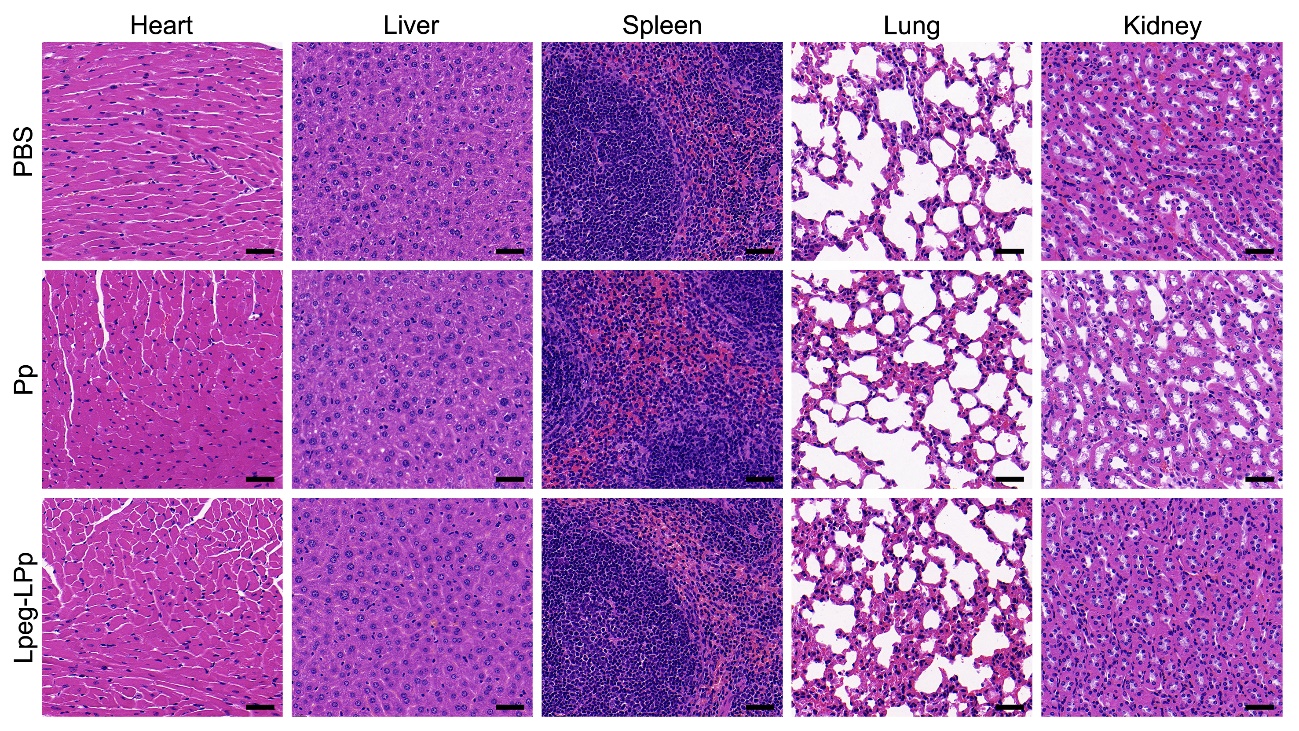


**Figure S10.** H&E staining of main organs harvested from Pp and Lpeg-LPp treated mice in comparison to PBS treatment. Scale bars, 40 μm.


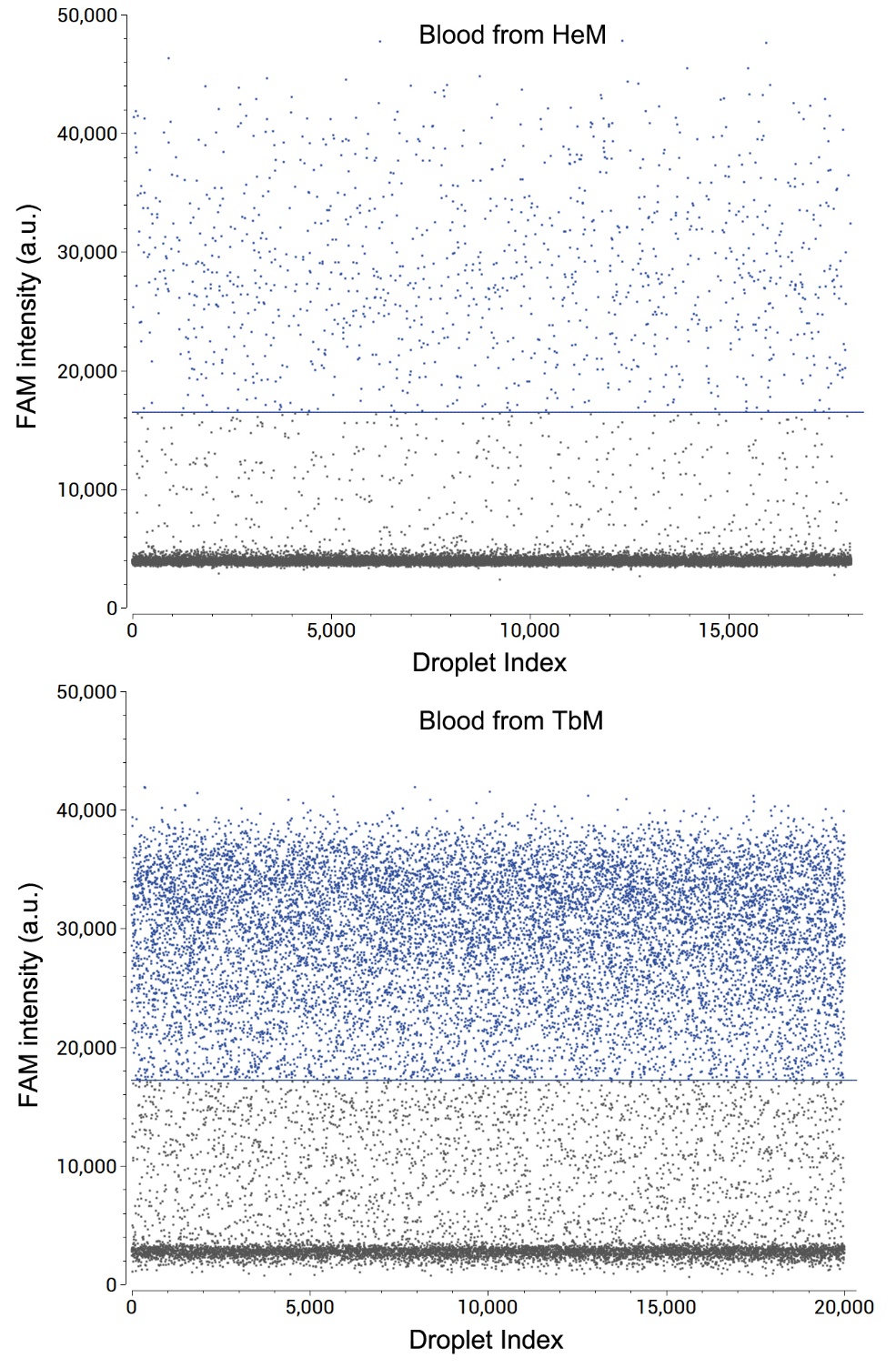


**Figure S11.** Representative results showing the quantitative analysis of KRAS(G12D) mutation in the serum ctDNA using ddPCR.


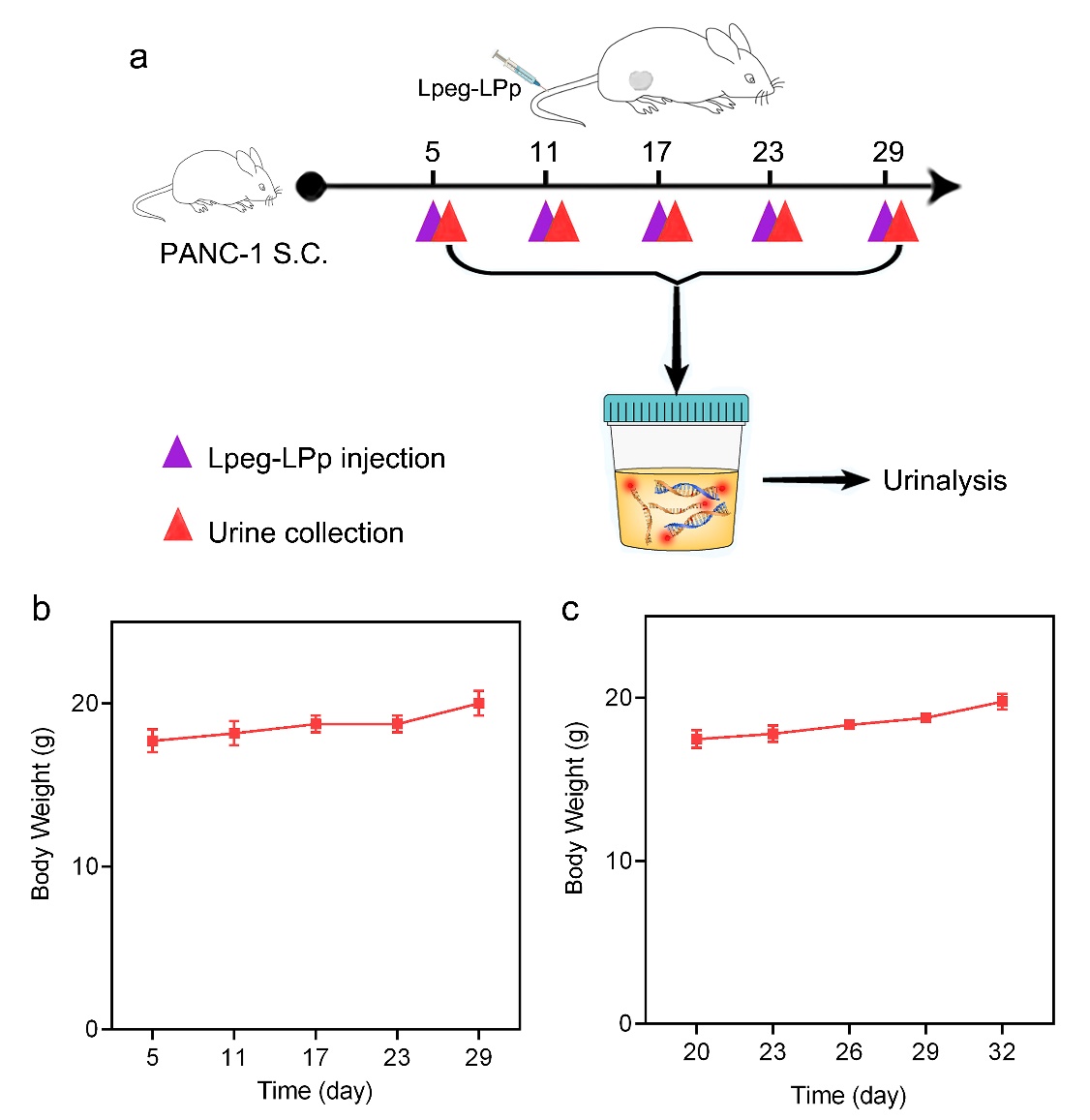


**Figure S12.** (a) Experimental design for urinary monitoring of tumor progression using Lpeg-LPp (Pp: 100 nmol/kg). BALB/c nude mice were inoculated subcutaneously with PANC-1 cells, then the tumor sizes were recorded and urine samples were collected at indicated time post- injection. Body weight of mice over the course of (b) tumor progression and (c) prognosis monitoring. Data are presented as means ± s.d. (n = 3).

Table S3. The advantages and limitations of conventional and state-of-the-art techniques for ctDNA detection.

| **Technique** | **Advantages** | **Limitations** |
| --- | --- | --- |
| PCR | High-throughput;  Multiplexed;  Ultrasensitive | Blood sampling and preanalytical processing required;  Limited specificity;  Time-consuming |
| DNA sequencing | High-throughput;  Highly sensitive;  Highly specific and reliable | Blood sampling and preanalytical processing required;  Time-consuming;  Expensive |
| Electrochemical sensing | High sensitivity;  Fast and on-line analysis,  Cost-effective | Blood sampling and preanalytical processing required;  Sensor and chip preparation is not easy |
| SPRI | High sensitivity;  High level of integration;  Cost-effective | Blood sampling and preanalytical processing required;  Preparation of nanoparticles and chip is complicated |
| Fluorogenic detection | Short measurement time;  Easy to handle;  Small size of the instrument | Blood sampling and preanalytical processing required;  Low sensitivity |
| Colorimetric assays | Excellent commercial potential; Easy to handle;  Cost-effective | Blood sampling and preanalytical processing required;  Low sensitivity |
| **Our nanoreporter** | No need for blood sampling and preanalytical processing;  Highly specific;  Easy to be prepared;  Easy to handle; Cost-effective | Sensitivity is limited;  Systemic administration is required |

**Abbreviations:**

**Pp:** γPNA oligomer for specific KRAS(G12D) mutation sensing.

**LPp:** The duplex prepared by hybridizing Pp with linker DNA.

**Lpeg-LPp:** The nanoreporter prepared by functionalizing DBCO-bearing LPp and DSPE-PEG (2 kDa) onto 8-arm poly(ethylene glycol) nanocore.

**Lpeg-LPp^on^:** The always-on nanoreporter without BHQ3 labeling on linker DNA.

**Lpeg-LPs:** The nanoreporter engineered for KRAS(G12S) mutation detection.

**Ppc:** The control γPNA oligomer of Pp.

**LPc:** The duplex prepared by hybridizing Ppc with linker DNA.

**Lpeg-LPc:** The nanoreporter prepared by functionalizing DBCO-bearing LPpc and DSPE-PEG (2 kDa) onto 8-arm poly(ethylene glycol) nanocore.

**12DM:** The ctDNA with proto-oncogene KRAS(G12D) mutation.

**WT:** The wild type ctDNA without mutation.

**CDNA:** Control DNA strand with random nucleobase sequence.

**TbM:** Tumor-bearing mice.

**HeM:** Healthy mice.
